# A Comparative Analysis on Blockchain versus Centralized Authentication Architectures for IoT-Enabled Smart Devices in Smart Cities: A Comprehensive Review, Recent Advances, and Future Research Directions

**DOI:** 10.3390/s22145168

**Published:** 2022-07-10

**Authors:** Usman Khalil, Owais Ahmed Malik, Mueen Uddin, Chin-Ling Chen

**Affiliations:** 1 School of Digital Science, Universiti Brunei Darussalam, Jalan Tungku Link, Bandar Seri Begawan BE1410, Brunei; owais.malik@ubd.edu.bn; 2 Institute of Applied Data Analytics, Universiti Brunei Darussalam, Jalan Tungku Link, Bandar Seri Begawan BE1410, Brunei; 3 College of Computing and Information Technology, University of Doha for Science and Technology, Doha 24449, Qatar; mueen.uddin@ubd.edu.bn; 4 School of Information Engineering, Changchun Sci-Tech University, Changchun 130600, China; 5 Department of Computer Science and Information Engineering, Chaoyang University of Technology, Taichung 41349, Taiwan

**Keywords:** smart device, smart city, blockchain, decentralized ledger technology (DLT), cyber-physical system, internet of things, IoT, security, authentication

## Abstract

Smart devices have become an essential part of the architectures such as the Internet of Things (IoT), Cyber-Physical Systems (CPSs), and Internet of Everything (IoE). In contrast, these architectures constitute a system to realize the concept of smart cities and, ultimately, a smart planet. The adoption of these smart devices expands to different cyber-physical systems in smart city architecture, i.e., smart houses, smart healthcare, smart transportation, smart grid, smart agriculture, etc. The edge of the network connects these smart devices (sensors, aggregators, and actuators) that can operate in the physical environment and collects the data, which is further used to make an informed decision through actuation. Here, the security of these devices is immensely important, specifically from an authentication standpoint, as in the case of unauthenticated/malicious assets, the whole infrastructure would be at stake. We provide an updated review of authentication mechanisms by categorizing centralized and distributed architectures. We discuss the security issues regarding the authentication of these IoT-enabled smart devices. We evaluate and analyze the study of the proposed literature schemes that pose authentication challenges in terms of computational costs, communication overheads, and models applied to attain robustness. Hence, lightweight solutions in managing, maintaining, processing, and storing authentication data of IoT-enabled assets are an urgent need. From an integration perspective, cloud computing has provided strong support. In contrast, decentralized ledger technology, i.e., blockchain, light-weight cryptosystems, and Artificial Intelligence (AI)-based solutions, are the areas with much more to explore. Finally, we discuss the future research challenges, which will eventually help address the ambiguities for improvement.

## 1. Introduction

**Figure 1:**
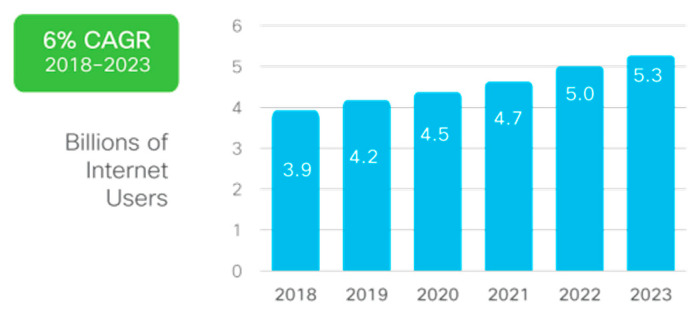
Growth Projection in Internet Users from 2018 to 2023 [1].

An exponential number of smart devices connecting to the internet with every passing day results in a network of low-powered devices that communicate with each other. Communication with/without a miner or server makes it possible to create a device-to-device (D2D) or a machine-to-machine (M2M) network [1]. On the other hand, the internet has become a global arena for connecting these smart devices, yet exponential growth has been observed in the number of connected smart devices. According to the recent Cisco Annual Internet Report (2018–2023), nearly two-thirds of the global population will have internet access by 2023 which means more devices may be connected to the internet in the future. The report projects approximately 5.3 billion total internet users, i.e., 66 percent of the global population, by 2023. It estimates a rise of 51 percent for the worldwide population noted in 2018 [1], as depicted in Figure 1.

**Figure 2:**
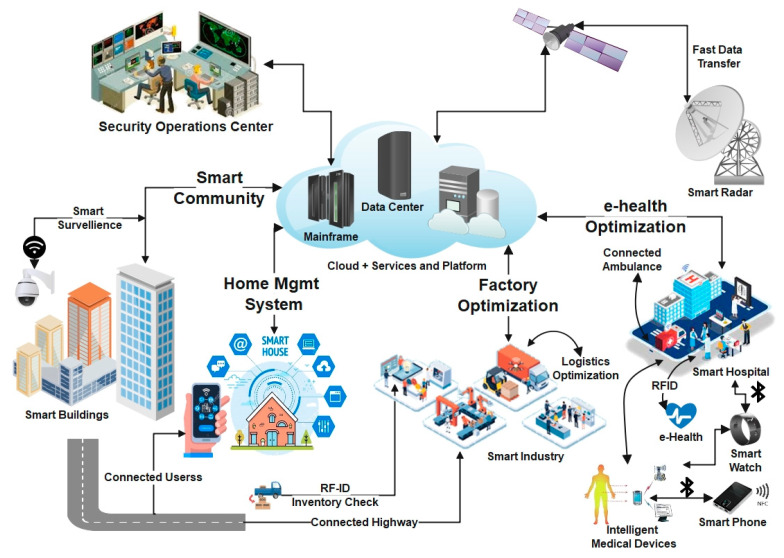
Generalized Smart City Architecture.

These IoT-related objects and processes have been developed based on the traditional transmission control protocol/Internet protocol (TCP/IP) stack-based internet. They are not designed for such a huge number of connected devices. It requires robust solutions that may provide the foundation for its implementation and integration. Due to the underlying network models, the increasing number of smart devices inherits the issues concerning privacy and security of the connected smart devices and the network itself [2]. These devices play an important role in every domain as they represent the edge of the network where real-time data collection is carried out in cyber and physical space. The wide adoption of these smart devices has led to the concept of smart cities, where many smart devices operate in different IoT networks. It supported the realization of other cyber-physical systems such as smart houses, smart parking, smart buildings, smart healthcare, smart retail, smart transportation, smart waste management, smart grid, smart agriculture, etc., as depicted in Figure 2 [3].

These IoT-enabled smart devices operating in their respective domains would eventually lead to the concept of a smart planet where all systems will be interoperable and interconnected. The edge of the network connects these smart devices, such as sensors, aggregators, and actuators that can operate in the physical environment and collect the data, further used to make informed decisions through actuation. Since these devices operate at the edge layer, they are referred to as edge nodes and are typically low-powered and responsible for sending a specific piece of information. The edge nodes that work under an IoT infrastructure are IoT devices [4].

### 1.1. Enabling Technologies

These smart devices utilize enabling technologies based on the type of networks, i.e., Wireless Sensors Networks (WSNs), fifth/sixth-generation technologies (5G/6G), Cloud Computing, Fog Computing, etc. [4]. The connectivity provided through the traditional network technologies such as Wireless Fidelity (Wi-Fi), Radio Frequency Identification (RFID), Bluetooth (BT), Near Field Communication (NFC), etc., supports data collection and transfers, as depicted in Figure 2. Since the smart city architecture also relies on the conventional internet supported by communication and transmission technologies for data collection and transmission, respectively, it is evident that it may inherit security and privacy challenges.

Here, the security of these devices is immensely important, specifically from an authentication standpoint, as in the case of unauthenticated/malicious assets, the whole infrastructure would be at stake. Many researchers in academia and industries have proposed different methods to secure these smart devices and the data generated by them. Considering these issues, we will be reviewing the literature focusing on authentication mechanisms for IoT-enabled smart devices in smart cities.

### 1.2. Related Surveys

Several surveys discuss the security challenges posed to IoT-enabled smart assets in a smart city context. The authors discuss the IoT authentication issues in [5], providing a wide range of authentication protocols proposed in the literature. Using a multi-criteria classification, they compare and evaluate the proposed authentication protocols, showing their strengths and weaknesses in multiple CPSs and identifying several requirements. The open issues may be considered while developing new authentication schemes for IoT networks and applications. The authors in [6] identify several key technical challenges and requirements for the IoT communication systems based on privacy, security, intelligent sensors/actuators design, low cost and complexity, universal antenna design, and friendly smart cyber-physical system design for its deployment. Finally, the authors present challenges in cyber-physical communication system deployment and related issues in implementing an efficient and effective IoT communication system. A comprehensive survey has been presented in [7] on cyber-physical systems (CPSs) concerning applications, technologies, standards, and related security vulnerabilities, threats, and attacks. It further leads to identifying the key issues and challenges within this domain

Additionally, the existing security measures have been discussed and evaluated to further strengthen the identification of limitations. Various security aspects, services, and best practices ensure resilient and secure CPS systems. The survey focuses on the CPSs that face challenges regarding security services, authentication, and authorization with suggestions and recommendations.

Blockchain (BC) technology’s evolution considering constituent technologies, consensus algorithms, and blockchain platforms have been presented in [8]. The authors discuss the security issues for smart cities and critically evaluate various smart applications enabled by blockchain-enabled solutions. An implementation based on a real-world blockchain scenario has been presented as a case study to strengthen the review further. The review presents the key needs for BC integration in smart cities and the research gaps from an improvement point of view. A systematic review of Internet of Things (IoT)-based smart cities and blockchain (BC) has been presented provided with statistical analysis in [9]. The authors discuss the distributed nature of BC, which has been adopted by many businesses, posing challenges in IoT-based smart cities. Since IoT has influenced modern society and industry, it poses some security and privacy susceptibilities. The authors present the systematic approach to identify the significant mechanisms and investigations regarding security in the IoT and BC in smart cities. The analysis shows that BC integration provides robust privacy, security, distributed storage, transparency, and trust, a dire need for IoT-based smart cities.

The review in [10] focuses on blockchain as a potential technology that can provide a robust security mechanism for smart cities. The authors present the state-of-the-art blockchain technology to solve the security issues of smart cities. It is due to its underlying properties of audit-ability, transparency, immutability, and decentralization. The authors suggest adopting blockchain in various smart communities such as healthcare, transportation, smart grid, supply chain management, financial systems, etc., which presents the future research directions of the survey. The authors in [11] emphasize the need to adopt security mechanisms that can handle the rising security issues that the ICT community will face in transitioning from the conventional way of living to the interconnected world of smart cities.

The review of blockchain models has been suggested as the interaction of blockchain with smart contracts, and their application in e-governance will support gaining robust security and privacy. The review suggests the improvement and additions of new scripts that might be able to assist the further improvement of this challenging and overly ambitious venture. The review in [12] presents an overview of layered IoT architectures and associated attacks. The mechanisms that provide the security solution to the security issues have been discussed, with the limitations posed in the same direction. The survey reviews the existing security mechanisms for protecting the IoT infrastructure and the restrictions and regulations of the current security methods. Several open research challenges associated with IoT technology have also been discussed for better understanding. The literature survey in [13] identifies the components of the smart city to realize the concept. The real-world implementations and statistical analysis are discussed, keeping in view the smart cities context. Since smart cities face serious challenges and issues due to enormous data processing demands and heterogeneity of smart assets, a review of those future research challenges has been identified, describing the opportunities for improvements.

The authors in [14] present current challenges of IoT and blockchain while an analysis of the potential advantages of both has been evaluated. The review of the available blockchain platforms and disruptive applications in this area has been highlighted to address these challenges. The authors in [15] discuss the characteristics of blockchain technology, focusing on the integration of distributed ledger technology in smart cities. A blockchain-based conceptual architecture explains security using a possible use case study. Additionally, a real-world blockchain-based smart city case study discussed several imperative research challenges.

A conceptual model of IoT applications for enterprises has been presented in [16] by identifying IoT applications in monitoring and control, big data and business analytics, and information sharing and collaboration. The survey focuses on the investment opportunities and evaluation with NPV and real options. The challenges in the IoT applications deployment have been discussed for enterprises.

The review of the aforementioned related surveys is expanded to multiple domains. However, IoT-enabled smart devices have become an important part of the architectures, such as the Internet of Things (IoT), Cyber-Physical Systems (CPSs), Internet of Cyber-Physical Things (IoCPTs), and Internet of Everything (IoE). In contrast, these architectures constitute a system to realize the concept of smart cities and, ultimately, a smart planet. The literature has been reviewed, considering all the aforementioned architectural domains. Security services Confidentiality, Integrity, and Availability (CIA), including authentication, authorization, and audit (AAA), are of utmost importance to safeguard these smart assets; hence, we have taken the lead in providing an updated review of authentication mechanisms. This comprehensive review is a continuation of a review article [17] that discussed the newly proposed solutions based on blockchain for the authentication of IoT-enabled smart devices. In this article, however, a categorical and descriptive approach has been adopted by categorizing centralized and distributed architectures. This review compares the mechanisms by discussing the security issues in the authentication and identification of IoT-enabled smart devices. Apart from blockchain-based solutions, other solutions based on artificial intelligence and cryptosystem have also been discussed in future research directions. The review of the proposed schemes has been evaluated based on the robustness and weakness standpoints which will eventually help address the ambiguities for improvement in the future. However, converging authentication schemes based on centralized and distributed architectures provokes new challenges. The major contributions of the article are presented below.

- We explore and discuss smart city layered architectures for employing authentication schemes in various smart city scenarios.
- We review and analyze the existing security services and their related challenges and issues in smart cities.
- We provide insightful reviews and discussions on the early adoption of traditional state-of-the-art authentication schemes for IoT-enabled smart assets to reveal their full potential in smart cities.
- We present a comprehensive classification and detailed reviews of the latest authentication schemes for IoT-enabled smart assets in smart cities.
- Furthermore, we categorically reviewed, evaluated, and analyzed IoT-enabled authentication schemes based on centralized and distributed blockchain-enabled smart city architectures.
- We present and elaborate on an emerging concept of Blockchain-as-a-Service as a result of reviewing existing solutions and discussing the related challenges and issues in smart cities.
- We identified and discussed the pros and cons of existing authentication schemes in smart city architectures.
- Finally, we provide the recent advances and future recommendations for IoT-enabled authentication schemes in smart cities and conclude the paper in the final section.

### 1.3. Paper Organization

The rest of the paper is organized as follows. Section 2 elaborates on the layered architecture of the smart city, followed by Section 3, which discusses the adversaries on each smart city layer. Section 4 and Section 5 discuss the security services and issues involved in smart city architecture. Section 6 reviews the traditional state-of-the-art authentication architectures. Section 7 and Section 8 thoroughly review the newly proposed authentication schemes based on centralized and distributed protocols. Later, recent advancements are presented about blockchain-enabled solutions and blockchain-based cryptosystems in Section 9. Section 10 thoroughly discusses the artificial intelligence-based solutions provided with future research challenges. Finally, a concise conclusion is presented in Section 11 at the end.

## 2. Smart City Layered Architecture

**Figure 3:**
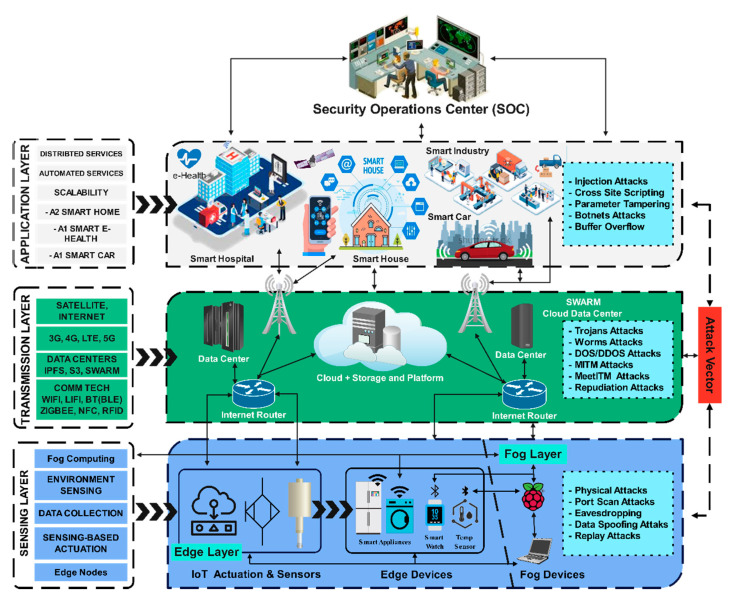
Generalized Smart City Layered Architecture.

The smart city architecture can be classified into layers based on the assets operating in a physical cyberspace environment that provides connectivity with the network for data flow, such as the internet. The data captured by the physical assets, i.e., sensors, aggregators, and actuators, are processed in the physical layer referred to as the sensing layer. The command-and-control work on the application layer defines the applications for the asset’s behavior at the physical layer. The network provides connectivity using communication and transmission technologies at the transmission layer. Though different researchers have different opinions [18], smart city architecture can mainly be divided into three-layered architecture, as depicted in Figure 3. The layers’ functions, issues, and weaknesses are further explored and discussed below in this section.

### 2.1. Application Layer

The application layer plays an important role in the applications defined with different functions for respective CPSs, such as application deployment for smart homes, smart hospitals, or smart cars. As depicted in Figure 3, this layer provides a path for the interaction using received information from the transmission layer. The commands are executed based on data received from the devices at the sensing layer [12,19]. The deployment is carried out in a smart city’s security operations center (SOC). It is the center point for service providers in a smart city architecture for utility companies connected to several applications located at different locations. The automated services provided at this layer may be centralized or distributed depending upon the nature and requirement of the CPS for its application and scalability.

### 2.2. Transmission Layer

As shown in Figure 3, the data from the application layer is transmitted through this layer. It is responsible for the communication among the devices between the upper and lower layers. These devices connect through the traditional network technologies already in use for transferring the collected data, i.e., Wireless Fidelity (Wi-Fi), Radio Frequency Identification (RFID), Bluetooth (BT), Near Field Communication (NFC), etc. In contrast, the transmission technologies such as 3G, 4G, LTE, 5G, internet, or satellite play an important role in data transfer and acts as the backbone for communication [4]. Routing devices such as routers and switches use communication and transmission technologies to route the data. In contrast, cloud computing platforms, internet gateways, firewalls, intrusion detection systems (IDS), and intrusion prevention systems (IPS) platforms facilitate smooth and secure data transmission. The datacenters by web servers such as Facebook, Google, etc., also function at this layer which may be centralized or distributed in nature, as in the case of Interplanetary File System (IPFS), Swarm, S3, etc.

### 2.3. Sensing Layer

Next to the transmission layer is the sensing layer. It comprises an edge and fog layer and deploys the edge and fog devices such as sensors, aggregators, actuators, and raspberry Pi/servers to achieve real-time data processing. Later, the collected data can be used to make informed decisions based on CPS requirements in a smart city, as depicted in Figure 3. For instance, actuating the lights to switch on/off, recording a video whenever any moving object is detected, or turning on/off any smart device whenever sensing the heat signatures triggers environment sensing that can be used to intimate the SOC for further action, etc.

## 3. Smart City Layered Adversaries

The smart city concept can improve the efficiency of the maintenance and replacement operations of the involved devices, keeping adversaries in view. The data transfer among the layer and devices is of utmost importance as data integrity and anonymity preserves the data being leaked. In contrast, user and device authentication prevent unauthorized access in case of an attack vector. This section further explores the smart city layers from an adversarial point of view, as discussed below.

### 3.1. Application Layer Adversaries

Since the user interaction is provided through the application layer, the attack vector finds it lucrative to exploit loopholes that are left unattended consciously or for a better end-user experience. As shown in Figure 3, the most common attacks at this layer are injection attacks, cross-site scripting attacks, parameter tampering, botnet attacks, and buffer overflow attacks, with details mentioned below.

#### 3.1.1. Injection Attacks

The smart city architecture is highly prone to malicious attacks that include different injection attacks such as code injection attacks, SQL injection attacks, code-reuse attacks, etc. However, injection attacks normally inject malicious code into an application to disrupt its functioning, exploiting untrusted resources [20,21,22].

#### 3.1.2. Cross-Site Scripting Attacks

These injection attacks introduce malicious scripts to the trusted web servers that molest benign websites. Also known as cross-site scripting (XSS) attacks, they share malicious scripts in the form of browser-side scripts to multiple users causing site crashes [12,23,24].

#### 3.1.3. Parameter Tampering Attacks

As the name suggests, it targets the tampering of parameters in the Uniform Resource Locator (URL) stored in the web server without the user’s consent. It causes the website to crash or fraudulently change the credentials in the Web page form field entered by a legitimate user. A group calling itself “New World Hackers” attacked Brian Krebs’ websites of famous American journalist and investigative reporters with a keen interest in pursuing cyber criminals the same way [25,26].

#### 3.1.4. Botnet Attacks

As discussed in Section 5.3, the manufacturers’ low concentration of security features in the IoT-enabled smart assets has uprooted the attack vector to target the devices according to the need. The flaws in security features such as easy-to-guess default login credentials, open ports, unencrypted and weak versions of SSL (v2, v3, and CBC mode) services, etc., give the attack vector an edge to turn these devices vulnerabilities to botnet attacks such as Ramnit (2015), Mootbot (2020), etc., causing the cost of IoT hacks for small US firms amounts to 13% of their annual revenue [27,28,29,30].

#### 3.1.5. Buffer Overflow Attacks

Also referred to as Transmission Control Protocol (TCP) SYN Flood or Hyper Text Transfer Protocol (HTTP) flood attacks, the attack vector exploits the TCP handshake process or SYN packets or seemingly legitimate HTTP GET or POST requests by constantly forwarding the requests to the server without any need to get a response. It creates a waiting buffer to overflow with increased fake requests in time to get a reply. Ultimately, the infrastructure may suffer application crashes that hinder the delivery of smooth and continuous services in smart cities.

### 3.2. Transmission Layer Adversaries

This layer can be targeted by obstructing the network resources and bombarding the fake data. It can lead to serious consequences such as distributed denial-of-service attacks (DDoS). As shown in Figure 3, the other types of attacks may be similar attacks, i.e., trojan attacks, worm attacks, denial-of-service attacks (DoS), or data can be spoofed by man-in-the-middle attacks (MITM), meet-in-the-middle attacks (MeetITM), and repudiation attacks. At the same time, one-way encryption schemes are best suited to hinder the attack vector [27,29].

#### 3.2.1. Trojan Attacks

Trojan horse refers to a software or set of codes designed to sit in the archive bits of the computer, i.e., fake antivirus Trojans. It is one of the famous malware that has been used to destroy and steal important information or perform the desired action on corporate networks (i.e., DDoS trojans) from an adversarial point of view. Examples of trojan horse attacks are MiniPanzer/MegaPanzer, Gh0st RAT (2009), Shylock (2011), TinyBanker (2016), and Banking Trojans (2020) [31,32,33].

#### 3.2.2. Worm Attacks

The worm attacks involve the injection of codes to the networks that target the operating system weaknesses to steal, modify, and delete data at the web client and the webserver. Examples of worm attacks have been discussed in Section 5.2 (such as Stuxnet, Flame, and Duqu, i.e., Code Red/Code Red II (2001), Nimda (2001), Triton (2017) [7,34,35].

#### 3.2.3. Denial-of-Service (DoS) Attacks

Section 5.1 and Section 5.2 discuss DoS attacks in smart cities that forbid authentic users from accessing devices and network resources. Typically, the devices and network resources are flooded with requests that cause the system to crash because of computation exhaustion. At the same time, it exists in different forms such as blackhole, teardrop, etc. [12,14,33,36].

#### 3.2.4. Distributed Denial-of-Service (DoS) Attacks

Distributed denial-of-service (DDoS) attacks are similar to those of the aforementioned DoS attacks; however, these attacks are more sophisticated. In these attacks, the service delivery is compromised to the resources such as network devices, applications, and even specific functions within applications. It may also exist in different forms such as ping-of-death, Smurf, and Black Energy series (BE-1, BE-2) [30,37,38,39].

#### 3.2.5. Man-in-the-Middle (MITM) Attacks

As the name suggests, the script is in the middle of communication between a legitimate user and an application. The man-in-the-middle attacks are known as benign scripts that spy on the online exchange of information packets transferred between a legitimate user and an application by mimicking one of the parties for the exchange of information, thus taking hold of important information for personal gains [2,12].

#### 3.2.6. Meet-in-the-Middle (MeetITM) Attacks

Contrary to the man-in-the-middle attacks, the meet-in-the-middle attacks focus on the cryptosystem that encrypts important information to provide data integrity and anonymity. It is also known as a plaintext attack as it focuses on getting hold of the information in plaintext encrypted by the encryption algorithms. Examples include Double DES or Triple DES, which can be cracked using brute force with 2^56^ space and 2^112^ operations from an adversarial standpoint [40].

#### 3.2.7. Repudiation Attacks

These attacks focus on manipulating the identification of new actions when applications or architectures do not prioritize the safety of the user’s track and log actions. These attacks mostly deal with the general data manipulation in the name of others, similar to spoofing mail messages. If this attack occurs, the data stored on log files can be considered invalid or misleading [41].

### 3.3. Sensing Layer Adversaries

The devices at the edge node are to be safeguarded in case of attacks, or the assets may be damaged or stolen. As depicted in Figure 3 and discussed below, the adversaries at the sensing layer, such as physical attacks, port scanning attacks, eavesdropping, and replay attacks, are the most common attack for data spoofing and checking the behavior of the environment in which they operate [42,43,44,45].

#### 3.3.1. Physical Attacks

These attacks refer to the physical damage to IoT-enabled assets and related resources. However, different cyber-physical systems (CPSs) in a smart city may implement various forms of protection, i.e., power grid, power plants, base stations, etc., are well-protected. These CPSs do not allow weak physical security, or the assets may be destroyed, stolen, or sabotaged. An example may be Smart Meters, which must be tamper-resistant by relying on the outage or host-based intrusion detection. Physical attacks are inevitable, except the impact may be reduced [12,21].

#### 3.3.2. Port Scanning Attacks

The data transmission usually takes place using different ports defined for specific purposes that utilize PAT (Port Address Translation) as one of the solutions on the hosts. These ports can easily be scanned using tools such as Advance Port Scanner and TCP port Scan, making them lucrative targets for attacks. The attack vector targets the ports by sending packets and gaining information for services and service versions to find the vulnerability of the host [2].

#### 3.3.3. Eavesdropping Attacks

Non-secure communication among the network resources and the devices is prone to eavesdropping attacks as it may contain sensitive information (i.e., passwords, usernames, phone calls, text messages, fax transmissions, or any other information relating to the smart city). Eavesdropping can be active and passive depending upon the nature of the information to be eavesdropped on [44,46].

#### 3.3.4. Data Spoofing Attacks

It consists of masquerading the identity of a trusted entity by a malicious unknown source. In this case, attackers can spoof sensors, for example, by sending misleading or inaccurate measurements to the control center [28].

#### 3.3.5. Replay Attacks

In these attacks, attack vectors eavesdrop on the transmitted packets to access the authentic information from the sender and receiver, also known as playback attacks. In a smart city context, it may include snooping on the information packets between PLCs, RTUs, and ICSs to disrupt the real-time operations and affect their availability causing delays in operations. The replay of the snooped authentic information may lead to modified data obstructing the normal working for time-critical operations [7,12].

## 4. Smart City Layered Security Services

The security and privacy of the data, devices, users, etc., are controlled by a framework known as the AIC triad, where Availability, Integrity, and Confidentiality play an important role in securing data communication in a smart city concept.

1. Confidentiality refers to protecting data from unauthorized disclosure means the information sent by the sender should be received by the correct recipient (user), which ensures message confidentiality.
2. Integrity refers to protecting data from unauthorized alterations and changes, and the content should be transmitted untampered, which ensures message integrity
3. Availability refers to protecting data from unauthorized access by transmitting data to the authentic user to ensure message availability.

Apart from the AIC triad, other factors have caught the attention recently, such as Possession or Control, Authenticity, Authorization, Audit (AAA), and Non-Repudiation [47].

1. Authentication is an important process ensuring the identity of the assets or objects. In contrast, it is also of immense importance for a CPS in a smart city to have all the assets identified and authenticated to mitigate the attack vector within a system.
2. Authorization works side-by-side with authentication. Once the assets are authenticated, these assets need the authorization to carry out a specific task, which means not all authenticated assets will be able to carry out all tasks rather than authorized tasks only.
3. Non-repudiation is the ability to demonstrate that a task or event has occurred by the object and cannot deny the authenticity of a specific data transferred [47,48].

Authentication and secure communication need the security solution for smart city-based CPS and how those can quickly be added to the IoT and embedded devices to ensure they can be protected from cyber-attacks. Whenever an IoT device in a CPS connects to the network, it is subjected to many security risks such as eavesdropping, man-in-the-middle (MITM), and unauthorized access or control, especially when it connects internet, as depicted in Figure 3. However, the devices in the smart city are all about connectivity, which means all the devices will be connected.

## 5. Smart City Layered Security Issues

Though the internet provides a platform for connectivity and creates an ecosystem where all the assets will communicate (D2D, M2M, etc.), it is fundamentally unsafe. Many specialists and researchers believe that “IoT is going to hit us hard if we’re not doing anything about it.” [49]. For every service, every process, and API, the attack vector would always be looking to find the loopholes to break through the various layers of security mechanisms and protocols. A review of security issues keeping in view the same has been discussed in this section, leading to the current evaluation of security issues in smart city infrastructure.

### 5.1. Security Issues in Internet Infrastructures

As discussed in Section 1, the use of IoT-enabled smart devices is not limited to any specific field or industry. The objects associated with them have become more intelligent and smarter. It causes these devices to be prone to security issues exploited by the attack vectors on different communication layers that have been categorized as physical attacks, physical and link-layer attacks, also known as sensing layer attacks, network layer attacks, application-layer attacks, and multilayered attacks [3]. If compromised, these smart devices become the mainstream arena for cyberattacks to exploit the vulnerabilities of the devices and deploy IoT botnet attacks that cause major issues in the internet core for data transmission.

An example, in this case, is MIRAI BOTNET. The group utilized “Mirai” to scan the internet and found the IoT-enabled smart devices vulnerable to a cyberattack with their default login details. The assets were hacked and were used to attack a huge botnet that chocked half of the internet in the United States and was named “the most serious distributed denial-of-service (DDoS) attack in the history of the country [50]. Attacks like DoS and DDoS jammed the network flow [27], while the increasing number of IoT networks have faced challenges based on the security and privacy of the smart devices and data generated [51].

### 5.2. Security Issues in Cyber-Physical Systems

Security is critical for building IoT-enabled smart devices in a smart city, including secure communication and strong authentication for users and devices. In context to CPSs such as fields of smart grid, health monitoring, smart vehicles (UAVs, UGVs), process control, oil, and gas distribution, transportation system, etc., more complex large-scale systems have been developed and deployed at the industry level, such as Supervisory Control and Data Acquisition system (SCADA) [52,53]. These CPSs provide command, control, communications, computer, intelligence, surveillance, and reconnaissance (C4ISR) facilities, considered the backbone of any industrial architecture [53].

#### 5.2.1. Security Issues in Industrial Cyber-Physical System

As detailed earlier, the customer premises equipment (CPE) in cyber-physical systems (CPSs) generates data (sensors) which is crucial to making informed decisions (actuators) or decisions for corrective measures to resolve operational issues. In contrast, implementing these devices in a corporate system such as supervisory control and data acquisition (SCADA) is critical. Here, authentication becomes of immense importance from an adversarial point of view that may cause serious damage to the CPS, as in the case of the industrial programmable logic controllers (PLCs). The automated engagement of electromechanical processes for controlling the machines and industrial processes such as separating nuclear material from the centrifuges is performed. In case of a data breach, wrong data fueling may cause serious damage to the overall system or, in worst-case scenarios, be destroyed, causing a system halt [53]. A similar kind of security breach was reported in 2007. The Iranian Nuclear Program was hit by the Stuxnet virus, which works by propagating information across the network and the USB sticks [35]. The virus compromised Iranian PLCs, collecting data on industrial systems, and caused the fast-spinning centrifuges to tear apart [53]. According to Reuters, an asset protection US-based company, “Target,” was breached via the network to access the embedded devices with impunity. It caused a serious security risk to the data breach that hit 40 million payment cards data breach in the year 2013 [54]. The cyber-attack on the German steel plant in 2014 caused significant damage. The attack vector accessed the corporate network and moved unilaterally throughout the control network or operation network without any operational defenses [55].

#### 5.2.2. Security Issues in Health Care

In the case of the healthcare CPS, the issues related to the weak security in the wireless embedded medical devices such as pacemakers and insulin pumps (which record the patient details and treat the respective patients accordingly) may leak the patient’s critical health information. In case of false data injection, the results may be fatal. There had been major adversaries in the past where the vulnerabilities in the smart assets were exploited. A report was released on 29 December 2016, by the U.S. Food and Drug Administration (FDA) about the smart devices currently available in the market. It mentioned the issues related to the network security in wireless embedded medical devices such as pacemakers and insulin pumps, which could leak the patient’s critical health information [2,56]. Here, the authentication of connected devices has to be ensured while sending data to the corresponding storage devices, which is critical as far as the patient is concerned [2,51,56].

### 5.3. Security Issues in IoT-Enabled Smart Devices

Another factor is the manufacturers’ low concentration of security features in the CPE, such as easy-to-guess default login credentials, open ports, unencrypted and weak versions of SSL (v2, v3, and CBC mode) services, self-signed or expired security certificates, etc. Thus, it becomes an easy target for the attack vector who exploits these features to attack the system as a botnet that happened a couple of times in the past. The manufacturers of these devices left unattended authentication and access control schemes which increases the chance of the attack vectors’ exploitation.

In [27], an analysis of the ten most popular consumer IoT devices showed 250 susceptibilities concerning outdated operating systems, open telnet ports for making a remote connection to the device for exploitation, and weak encryption protocols configuration for data transmission. Authors in [57] evaluated 45 IoT devices from well-known vendors such as Amazon (Echo, Fire TV), Apple (HomePod, TV), D-Link (Cloud Camera), Google (Home, Home Mini, OnHub), Philips (HUE), TP-Link (Wi-Fi Bulb, Wi-Fi Plug), Samsung (Smart Things, Smart TV) and Logitech (Harmony), etc. They found almost the same kind of issues together with 84 running services. Secure Shell (SSH), Universal Plug n Play (UPnP), HyperText Transfer Protocol (HTTP webserver), Domain Name System (DNS), Network Virtual Terminal Protocol (Telnet: A service for remote connection to devices), Real-Time Streaming Protocol (RTSP) and custom services to name a few, while 39 issues related to those services were found. Though many state-of-the-art authentication and authorization mechanisms have been proposed for devices in smart cities, most are centralized and offer high communication overhead, which results in higher energy consumption.

### 5.4. Security Issues in Heterogeneous IoT-Enabled Smart Devices

Different manufacturers and vendors produce IoT-enabled smart devices that use various security and communication protocols to connect to the same IoT infrastructure. Since these heterogeneous devices connect in the same CPSs, making a heterogeneous infrastructure for data transfer and communication mechanisms at respective layers. It also causes the infrastructure to generate a huge amount of heterogeneous data. The authors in [3] also discuss the IoT infrastructure regarding the heterogeneous data generated by the heterogeneous things (IoT devices). The collection of this data poses an open challenge because of its volume and nature. It is important to take care of this data as hackers can easily hack it from IoT assets and later use it to manipulate the devices, such as in the case of Botnet attacks.

## 6. IoT-Enabled Smart Device Authentication Architectures in Smart Cities

As discussed earlier, the issues in a smart city can also be put into fundamental security traits categories, i.e., Authentication, Authorization, and Audit (AAA). It further classifies the security services into Confidentiality, Integrity, and Availability (CIA), whereas user confidentiality and authentication aspects have been explored. For any CPS in smart city infrastructure, the authenticity of users and customer premises equipment (CPE), i.e., sensors and actuators, are major concerns. With the rapid increase in usage and low concentration on the security and privacy details of the devices, challenges have been evident, pushing the need for solutions that could address these security issues.

Since this paper focuses on IoT-enabled smart device authentication schemes in a smart city, the upcoming section discusses the review of traditional state-of-the-art authentication mechanisms already deployed, followed by the newly proposed authentication mechanisms. A categorical approach has been opted to discuss an up-to-date survey of the conventional and freshly proposed authentication schemes based on centralized and distributed architectures.

### 6.1. State of the Art Authentication Models

**Figure 4:**
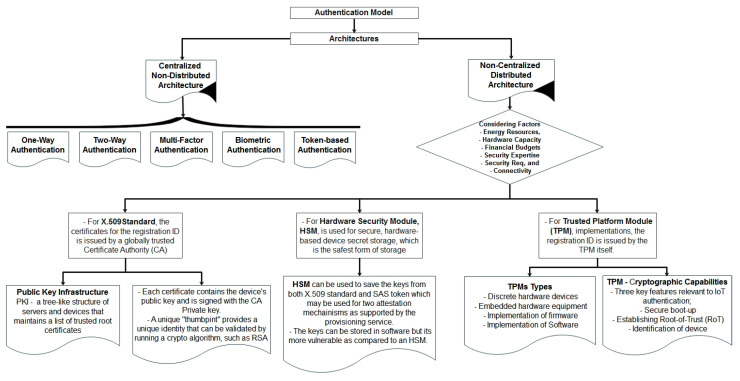
Authentication Model.

The authentication of CPE is as crucial as other CIA traits. The schemes would force legitimate users to access the resources; otherwise, the whole system would be at stake. As shown in Figure 4, the state of the art authentication model is based on authentication architecture with two main categories, i.e., centralized/non-distributed and non-centralized/distributed authentication models. The traditional authentication mechanisms have been developed on mostly centralized architecture where resourceful machines play an important role in providing authentication services. The attack vector always finds ways to intervene in the security protocols. That is where the additional authentication protocols come in handy such as OpenID [58], Security Assertion Markups Language (SAML 1.1/2), Fast Identity Online (FIDO), or Open Authorization (OAuth) [59,60,61]. At the same time, the implementation relies on trusted third-party (TTP) solutions. OAuth is the most adopted solution because it is one of the most powerful open authorization solutions available to API developers today. Its powerful functions can be utilized to protect the vast Internet of Things. Though it provides a strong and single sign-on (SSO) authentication mechanism, it also poses threats to its centralized architecture [62]. The centralized/non-distributed mechanisms, as depicted in Figure 4, involve the following authentication mechanisms that have been used traditionally and are discussed as under.

#### 6.1.1. Single-Factor Authentication (SFA)

SFA, referred to as One-Way Authentication, provides primary authentication such as password, secure PIN, PIV card, etc.

#### 6.1.2. Two-Factor Authentication (2FA)

2FA, referred to as Two-Way Authentication, provides one-time authentication such as a one-time password, secure PIN, registered device, etc. [38,63,64].

#### 6.1.3. Multi-Factor Authentication (MFA)

MFA depends on two to three factors that provide authentication, such as password, secure PIN, texting OTPs to users’ phone numbers and magic links sent via SMSs or emails, locational info, etc. In all the password-protected environments, protocols such as PAP and CHAP are used, which provides sufficient usability for the authentication mechanism [65,66].

#### 6.1.4. Biometrics

Biometrics includes biological traits, such as fingerprint ID and face shapes for user authentication. In 2011, a fingerprint scanner was introduced to the Motorola ATRIX Android smartphone, and since then, it has been commonly used for user authentication for device logging or completing digital purchases [31,67].

#### 6.1.5. Token-Based Authentication

It is a protocol used to authenticate the users to verify their identity based on the unique token received after verification of the credentials. These tokens are then used to access websites, applications, or any protected resource by mitigating the hassle of repeatedly re-entering the credentials. The token-based authentication mitigates the risk of stolen authentication factors as tokens are protected against misuse. It does not utilize system processing power like a password-based mechanism [68,69].

In smart city infrastructure, the authentication of the IoT-enabled smart devices in a cyber-physical system is immense. Though it is an important yet hard-hitting decision to choose the relevant model for authentication, the factors affecting the overall network’s performance would be at stake. The performance affects energy resources, hardware limitations, budgets, security expertise, security needs, and connectivity.

As depicted in Figure 4, the distributed security model works on certificate-based authentication, hardware-based authentication with secret storage, and trusted platform-based authentication solutions [70].

#### 6.1.6. Certificate-Based Authentication

This authentication mechanism utilizes X.509 Standard, one of the most implemented and preferred choices that rely on the certificates for authentication and authorization. These certificates are used for ID registration, issued by a globally trusted Certificate Authority (CA). It is referred to as Public Key Infrastructure (PKI), which consists of a tree-like structure of servers and devices that maintain a list of trusted root certificates. The certificates contain the device’s public key and are signed with the CA’s private key. A unique “thumbprint” provides a unique identity that can be validated by running a crypto algorithm, such as RSA [71].

#### 6.1.7. Hardware Security Module

Further shown in the figure, the other type of authentication is provided by hardware-based devices. It offers the Hardware Security Module (HSM), which secures the hardware-based device’s secret storage and is considered one of the safest forms of secret storage. HSM can save the keys from X.509 standard and SAS token, which may be used for two attestation mechanisms supported by the provisioning service. The keys can be stored in software, but it is more vulnerable than an HSM [72].

#### 6.1.8. Trusted Platform Module

The Trusted Platform Module (TPM) implementations include the registration ID issued itself by its hardware. TPMs come in several forms, such as discreet hardware devices, embedded hardware equipment, firmware, implementation of software, etc. TPM has several cryptographic capabilities, while a few key features may be quite relevant to IoT authentication, i.e., secure boot-up, establishing the root of trust (RoT), and identification of the device [42]. The aforementioned authentication schemes have traditionally been used; however, new authentication mechanisms have been proposed.

Following the categorical approach, an up-to-date survey of the freshly proposed authentication schemes based on centralized and distributed architectures discusses the security issues in the authentication of IoT-enabled smart devices. The review of the proposed techniques has been evaluated based on the robustness and weakness standpoint, which will eventually help address the ambiguities for improvement in the future.

## 7. Authentication Schemes Based on Centralized Architectures in Smart Cities

The resources are shared over the interconnected network in centralized or non-distributed authentication and authorization systems. Still, the decisions are centrally controlled by a miner machine such as a server in the case of client-server computing. Here, any service that needs the authorization to carry out a specific task gets routed to the central server, which approves if all the decision keys meet the criteria or disapprove otherwise. Most systems worldwide have implemented centralized mechanisms as a safety precaution. The server must approve every request to stop illegitimate system use [73,74]. The authentication schemes based on centralized architecture have been presented in this section, and an analysis of security issues posed by these mechanisms for future research goals.

### 7.1. Smart Offices and Smart Houses

The Smart Offices concept has been discussed in [75] as efficiency, productivity, and facility are the key factors for improvement and efficient working. However, the IoT assets and other devices in a smart office environment pose cybersecurity challenges in a smart cities concept. Out of those cybersecurity challenges, continuous and non-invasive authentication mechanisms for all these assets are of immense importance; hence, the authors proposed designing and deploying a continuous and intelligent authentication architecture oriented to Smart Offices. The architecture is oriented to the cloud computing paradigm and utilizes Machine Learning (ML) techniques using a classification algorithm such as Random Forest to authenticate users according to their behaviors. The metrics such as Precision, Recall, and F1 Score have been used to evaluate the results. The classification evaluation of personal computers shows an average precision of 96.32%, an average recall of 90.00%, and an average F1 score of 92.70%. In contrast, the classification evaluation of mobile devices was noted with an average precision of 97.43%, an average recall of 96.20%, and an average F1-Score of 96.76%. The evaluation results are deemed good as far as identifying devices and differentiating users are concerned.

The authors in [57] proposed a methodology for analyzing security issues for IoT embedded assets commonly employed in smart homes. The methodology poses a centralized architecture based on cloud computing utilizing could endpoints for IoT assets communication, storage, and security issues such as IoT assets misconfiguration, weak authentication, vendors patch through device updates, etc. The proposed methodology utilizes the literature for home-based IoT to have the knack to mitigate the attack techniques, mitigations, and stakeholders. The authors analyzed 45 devices to identify neglected research areas and discuss the security properties, i.e., attack vectors, mitigations, and stakeholders. The evaluation was performed using Nessus scanner to access the devices and cloud endpoints, Kryptowire, MobSF, and Qark to assess the mobile applications, and Nessus Monitor, ntopng, sslsplit, and Wireshark to assess the network layer communication protocols. Yahoo Cloud Services Benchmark Test (YCSB) was used for testing, which modifies the YCSB workload module and data interface layer. The community has provided a public portal to share and contribute independent findings utilizing their evaluation data.

The ultimate goal of ubiquitous computing can be achieved if the IoT assets are used by embedding them into the environment, giving the user assets that would be internet-powered and smart enough to make informed decisions. The authors in [76] present a Smart Home System (SHS) that contains all sorts of smart entities necessary at home. The authors introduced an embedded chip, i.e., SoC (System on Chip), named iVision. It has been used to interact with the IoT assets such as Smart Wall and Smart TV based on Device Profile for Web Services (DPWS); however, the analysis shows the performance and latency issues. A stack of S3C6410 embedded boards mimics the grid to achieve realistic results at home. Real web services were deployed to run on a smart device such as an air conditioner, refrigerator, microwave oven, etc. Many probing messages made the network behave peculiar, while resolving detected drivers takes three more seconds per device, and probing ten or more devices without external noises will require 1300 ms. Additionally, context-awareness-driven architecture in a CPS was suggested to improve the quality of the service in a Smart Home system. It is quite high in latency for device identification in time-critical cyber-physical systems in a smart city context.

### 7.2. IoT Embedded Assets

To mitigate the exploitation of smart assets from physical attacks, IoT assets embedded with physical properties have been utilized to identify and eliminate the physical attacks on the device (such as impersonation attacks and side-channel attacks). The authors in [43] proposed a lightweight two-factor authentication mechanism that authenticates the IoT device and incorporates physical device properties. The proposed mechanism uses a defined function on the integrated chips (ICs) named Physically Unclonable Functions (PUFs), which implies the factors in the authentication mechanism. Moreover, the concept of reverse fuzzy extractor has been exploited to address the issue of noise during the PUF’s operation. The mechanism implies a centralized server which makes this architecture more central as the mechanism depends on the central authority (i.e., the central server) to store authentication data.

Moreover, the mechanism requires five exchanges of messages between the device and the server. The computation cost was calculated through a series of simulations of cryptographic operations using an Ubuntu virtual machine operating as a server. A single core 798 MHz CPU with 256 MB of Random-Access Memory (RAM) was used as an IoT device. The computation cost for IoT devices was noted at 2.92 ms for executing 5NH + NFE. Gen + 2NPUF operations while a server takes 3.39 ms to compute 5NH + NFE totaling 6.31 ms as the overall computational cost of the proposed scheme. The computational cost was observed to be significantly lower than previous authentication schemes.

As mentioned previously in [43], a similar approach but not server-dependent for the storage of the authenticated data, the authors in [77] describe a lightweight authentication mechanism that does not need storing the secret keys in a secure, centralized miner, i.e., server. This mechanism also defines authentication mechanisms based on Physically Unclonable Functions (PUFs), which describe the physical properties of the devices. This property helps the devices increase security from the device’s physical abuse in the case of adversaries. The authors present protocols for two scenarios, one that establishes IoT device and server communication and the other for D2D communication when two IoT devices want to establish a session. The proposed mechanism also required at least five exchanges of authentication messages and was evaluated in terms of computational complexity, communication overhead, and storage requirement. The computational complexity was lower as the complexity for both IoT-enabled smart devices and servers was the same, i.e., O (n + M(l)k). The media access control (MAC) length of 32 bits was used to calculate the communication overhead, which was noted as 42 bytes for message 2 in Protocol 1, while message 3 in Protocol 2 was 120 bytes. Comparatively, Protocol 1 was proved to be much more efficient than those mentioned in the literature as its longest message is approximately 68 bytes. The storage requirements for the proposed mutual authentication protocols do not impose storage of variables deemed necessary for device authentication rather stores critical data such as CRP pair (C_i_, R_i_) and the respective ID_i_ in the server; thus, storage requirements are very low.

### 7.3. Cryptosystem-Based IoT Authentication Schemes

The authors in [78] imply the project to find the minimum specifications required to implement secure authentication algorithms for different network specifications. They implement one of the most known asymmetric cryptosystem algorithms, i.e., the RSA algorithm for IoT-enabled smart devices. The focus was on the devices with single-core CPU performance of 100 MHz or below, i.e., IoT devices such as WSNs and RFIDs. For evaluation, the experiments were carried out on a Pi as a server machine; however, the CPU was restricted to 100 MHz one core and 1024 MB of RAM to mimic the resource constraint device properties. The authentication algorithm based on the RSA PKCS initiates by first signing, then verifying a message to authenticate the server, followed by verifying the client’s signature. The RSA algorithm’s performance was measured as the devices could authenticate about 37 per minute. The scenario requires the gateway to handle 50 RSA authentication attempts per minute, but with an average of 12.3 MHz, while the authentication process needs 1.6 s to handle one request. It restricts the device to handle 37 authentications per minute. The time is taken for sending and waiting for the client’s verification, and the client’s response was disregarded in the testing. The computational overhead would increase with reduced computational power, as in the case of smart devices, which may cause issues in device authentication.

The authors in [71] discuss a designed authentication mechanism based on the crypto algorithm, i.e., RSA, for a smart IoT environment over the air network using state-of-the-art industry standards. The mechanism provides the security services CIA (such as Confidentiality, Integrity, and Availability), including X.509 certificate, RSA-based Public Key Infrastructure (PKI), and challenge/response protocols with the help of proxy induced security service provider. They describe an innovative system model, protocol design, system architecture, and evaluation against known threats. Additionally, the implemented solution is designed as an add-on service for multiple other sensitive applications (smart city apps, cyber-physical systems, etc.). It needs the support of an X.509 certificate based on hard tokens to populate other security services, including confidentiality, integrity, non-repudiation, privacy, and anonymity of the identities. The authentication scheme was evaluated based on computation and communication cost comparison using 64-bits. The cryptographic one-way hash function and unique random number were 128-bits each. RSA parameters were 1024-bits, totaling 1024-bits each for RSA public/private key. The communication cost of the proposed system login phase was observed as 1024 + 128 + 64 = 1216-bits. The length of the messages during the authentication phase was observed as 320-bits. Thus, the total cost observed was 1216 + 320 = 1536-bits which was much more efficient than other schemes. The storage requirement was observed at 2432-bits, much lower than the compared schemes. The proposed scheme was also evaluated against known vulnerabilities, and detailed comparisons are provided with popular known authentication schemes.

A new Token-Based Lightweight User Authentication (TBLUA) for IoT smart devices has been proposed [68]. It is based on the token technique to enhance authentication robustness. A token-based user authentication scheme would be more secure since authenticating users based on a password mechanism is not feasible in smart city applications such as smart hotels and offices. Security evaluation shows token security, Perfect Forward Secrecy (PFS), etc., as robust mechanisms and remains a strong competitor among existing ones for user authentication in IoT environments. The authentication scheme was evaluated based on computation cost comparison, observed at 8 ms for the user, 18.2 ms for the GW node, and 3.5 ms for the smart device in the login and authentication phase. The communication cost comparison for elliptic curve cryptography (ECC)-based schemes with a security level of 160-bit was considered. The observed communication cost was 84 bytes for the user, 156 bytes for the GW node, and 44 bytes for the smart device, costing 284 bytes. The simulation results were promising and efficient comparatively.

### 7.4. E-Governance in a Smart City

A novel smart-card-based remote user authentication protocol for e-governance applications has been proposed in [79] during Citizen-to-Government (C2G) type of e-governance transactions. The authors discuss the risk factor of data transmission and device security issues in e-governance in a smart city scenario. The authors propose a lightweight, robust remote user authentication and key agreement protocol based on dynamic identity protocol in contrast to static identity protocol, which easily leaks information. The user’s identity is unique in every login; thus, even if the attacker records the ongoing communication and replays the messages, he fails to log in as a legitimate user. It employs timestamps to avert replay attacks. The protocol meets all security attributes and is resistant to all well-known attacks. The protocol has been proposed to keep in view the generation of the public and private keys from the server and stores on a Smart Card (SC), which can further be used for Registration, Login, and authentication purposes such as validating the user and the server. The proposed protocol has been simulated on On-the-fly Model-Checker (OFMC). The computational cost of the proposed mechanism has been represented by the notations (i.e., TE, TH, TM, and TS). TE denotes the time complexity for computing the exponential, TH denotes hash, TM denotes multiplication/division, and TS denotes symmetric decryption and encryption functions. The computation cost for the registration phase was noted at 3 T_H_, the login and authentication phase was noted at 10 T_H_ + 3T_E_, and password change was noted at 2 T_H_ with a total cost of 15 T_H_ + 3 T_E_. The proposed protocol is lightweight as it uses XOR and a one-way hash operation and takes minimum cost, i.e., negligible, compared to other operations. AVISPA (*Automated Validation of Internet Security Protocols and Applications*), a security analyzing tool, has been used to verify the internet security of the protocol, marking the protocol security as SAFE.

A similar approach in [79] for smart e-governance applications in smart cities has been proposed in [80] while the author addresses adversaries’ issues. In contrast, the communication between the applications and the smart city is carried out, and an advanced multi-factor user authentication scheme has been proposed to address this issue. The registered users receive a valid smart card accumulated with some parameters as confidential values needed to login into the system and contact the registered cloud server to avail themselves of the services. The author claims the nature of the scheme is lightweight and resistant to many network attacks. The mechanism is centralized and mainly depends on the central registration center (RC) that provides the parameters to the participants during the registration phase. Once the user registered, a valid smart card accumulated with some parameters as confidential values needed to login into the system and contact the registered cloud-server to avail the services. The Cloud Servers (CS) stores the secret values to authenticate each other (CS and User) by providing the parameters set for authentication. Once registered, the user does not need to register again via RC as both entities (CS and User) establish a secret session key to access the required services from CS as and when required. The proposed scheme is mainly partitioned into five phases as, a. The Registration, b. Login and Authentication, c. Password change, d. User revocation, e. Dynamic CSP addition.

Formal analysis was carried out for the proposed scheme based on the ROR model. A performance evaluation has been carried out, which shows the low computational overhead and proves that the scheme is efficient and applicable to e-governance applications in a smart city. The communication cost compared to earlier proposed schemes (computation cost ≈ 29.2121 ms, ≈13.3767 ms, ≈7.7447 ms, ≈2.2743 ms, ≈11.5891 ms) was much lesser, i.e., ≈2.2628 ms which shows that the proposed scheme results are more efficient. Regarding storage requirement, the storage required for the smart card was observed at 960-bits with 1056-bits costing in terms of communication deemed efficient among earlier proposed schemes. A formal verification using the AVISPA tool confirms the security of the proposed scheme.

### 7.5. Smart Grid in Smart City

The authors in [39] have proposed a hardware-assisted framework to secure communication among smart grid corporate networks and others. The smart grids utilize the power distribution network that actively interacts with consumer devices and back-end SCADA systems. The grid communication is insecure and susceptible to data compromise and network and physical device attacks. The framework provides security services such as the confidentiality and integrity of data between each node and authentication for the communicating parties. Furthermore, the presented framework is resistant to software-based remote hijacking as well as secret key extraction. As discussed in the authentication model and shown in Figure 4, the authors in the proposed framework also utilize digital certificates PKI infrastructure to bind the identity of nodes with the public-private key pair. In contrast, each node on the network is equipped with a TPM, which will act as a root of trust for the attached node. The TPM hardware provides a reliable trust anchor for the host machine in terms of performance overhead. An RSA implementation took 3.08 s to compute RSA encryption for 10 Kilobytes of data on a 32-bit Arduino microcontroller. NISTP256 Key generation process took 426 milliseconds, which is comparable to the key creation time of the TPM, while the signature generation process in the implementation took 451 milliseconds.

The authors in [81] propose a data-centric edge-computing infrastructure to host defense mechanisms in IoT clouds by integrating physical states in decentralized power-grid regions. The edge servers were equipped with knowledge of the power grids in different subsystems, and the proposed infrastructure was enhanced with security policies to defend against IoT-based attacks. The authors propose to deploy data-centric edge computing in IoT clouds and not power grid IT networks. Data-centric edge computing can function as a security middlebox. Each edge server can enforce a fine-grained security policy on connected IoT devices, such as “read” and “execute” permissions on measurements and control commands. The authors categorize the attack vector into two categories as Type A (control-related attacks (CRAs)) and Type B false-or-bad-data–injection attacks (FDIAs) and restrict the IoT devices in a CPS to read and execute commands permissions. Preliminary evaluations show promising results, indicating that edge servers can efficiently process physical data and manipulate network traffic at runtime. A single instance of an ONOS SDN controller to connect all switches in the simulated network was used in terms of communication overhead. It helped with low latency to configure the edge serves within 6ms while the capability to execute the proposed security policy in terms of goodput was also measured. The goodput varied from 4 to 7.5 Mb/s.

### 7.6. Physical Layer Authentication in Smart City

Other research studies focus on authentication schemes by exploiting the aspects of the physical layer such as cardiac inter-pulse interval (IPI)-based key exchange or the mismatched bits sequence generated by two communicating parties for reconciliation by exchanging information over a public channel. However, these techniques cause computational overheads, storage issues, and energy consumption [23,82,83,84,85,86]. Table 1 depicts a summary of centralized mechanisms with proposed authentication schemes while the issues with those schemes have also been discussed.

The proposed authentication schemes based on centralized architectures provide security; however, these mechanisms pose issues that are discussed below.

- The authentication mechanisms based on centralized architecture depend on the server machine for processing every authentication request, which poses a single point of failure and contact as far as the attack vector is concerned.
  - For token-based authentication schemes, the registration server (RS) is responsible for generating tokens on the internet in a centralized architecture which causes security and data privacy issues.
  - The client-server environment is prone to spoofing attacks with the exposed share session key.
- In the case of a hardware-based authentication mechanism, a hardware upgrade is required, which needs the manufacturer’s intervention and can be costly to implement.
  - PUFs, in this case, are the current trend that enhances the security of the assets from a physical standpoint as PUFs result from the manufacturing process of Integrated Circuits (ICs), which introduces random physical variations into the ICs microstructure, making it unique.
  - PUFs utilize the SRAM of the edge node, which increases the operational and computational overhead resulting in delayed operations.
- In the case of Smart Card-based authentication mechanisms, the communication between the applications and the smart city is carried out using an advanced multi-factor user authentication scheme which can be utilized for the smart e-governance applications in smart cities.
  - Other schemes utilize a central server-based XOR and hash operations for the password, user anonymity, mutual authentication, shared session key, and key freshness. It is an easy target for attacks such as replay, password guessing, message forgery, and brute force attacks.
  - The mechanisms are Smart Card dependent, as the public, private, and session keys are stored on it for registration, login, and authentication. However, a central registration center (RC) provides the parameters to the participants during the registration phase. In case of compromised session keys at RC, the whole system would be at risk of being attacked.
  - In case of card loss or theft, the system’s security would be at stake.
  - Sharing session keys using public and private keys infrastructure in centralized architecture would be a single point of failure and contact for the attack vector. The scheme depends on a centralized server to generate the public and private session keys.
- In the case of power grids and VANETs, the risk of compromised communication between the corporate network of the power grid and the edge server deployed in the cloud via the internet has to be considered.
  - The identification and authentication of devices and the system must be taken care of. In the case of an adversary, the power grid system behind the corporate network would be at risk.

## 8. Authentication Schemes Based on Distributed Architectures in Smart Cities

A non-centralized system, also known as a distributed system, consists of hosts interconnected by a network. The hosts here refer to the computers in an interconnected computer network. These hosts communicate with each other and other resources in the network, such as files and printers, with the help of network services provided by servers. These resources are shared over the interconnected network and can be used by distributed authorization system [73]. The authorization of the services runs for every software that needs it, meaning a copy of authorization and authentication results is saved locally by the resources. Every request acts as a local server, which requires no communication on the network layer [87]. The occasional synchronization with the central service makes it possible to have the updated decision (authorization and authentication decisions) at the edge nodes. It authenticates the hosts at the local level, contrary to the non-distributed system. Every decision request has to go to a centralized server machine for approval, thus making it a centralized system. This attribute of the distributed system poses security problems that are intricate and must be addressed in order to keep the system safe from any sort of attack vector. There are multiple reasons for having a distributed system, i.e., implementing authentication schemes on different hosts/nodes for IoT device authentication in a smart city context, and that is the reason the system is vulnerable to a variety of adversaries in the form of intruders as well as authentic users of the system. The specific trust assumption has to be studied and evaluated carefully to determine whether the use of a blockchain provides additional value. A review of such proposed authentication schemes for IoT assets has been provided with an analysis of security issues posed by these mechanisms for future research goals.

### 8.1. Blockchain-Enabled Smart Houses and Smart District

A case study for a blockchain-based smart home framework that deploys the IoT security model compared to a cloud-based smart home has been proposed [51]. The performance evaluation in terms of fundamental security traits such as confidentiality, integrity, and availability has been performed. The authors define the IoT infrastructure with various components for a smart home using the lightweight blockchain concept for security and privacy issues and discuss the implementation of various transactions and associated procedures. In a smart home, all IoT devices are connected to a miner connected to the blockchain, and a local storage device for storing the data from IoT devices has been introduced. The concept discusses how the blockchain public key authenticates the network traffic and provides security against DDoS and Link Attacks. The experiment showed that blockchain is a comparatively more reliable solution for a smart home-based IoT infrastructure in terms of security and privacy. At the same time, it proved to be quite manageable for low-energy devices.

The authors designed a smart district model in [85], which is the step necessary to build a smart city with the help of IoT smart assets using new technologies, e.g., Blockchain (BC). The authors suggest that the role of IoT devices and the BC-based approach would provide an efficient energy management system integrated into a platform. The authors present an architecture for automation, demonstrate how a smart district can be realized, and propose implementing the model to achieve an efficient energy management system, including energy, security, safety, environmental management, communication, information, etc.

Due to the increasing demand for home automation, smart users and houses would add to the concept of a smart city by integrating the smart grid, services, buildings, houses, and appliances that would interact and be connected for better Quality of Living (QoL). The smart district case study was carried out in Bergamo, Italy. The district was designed to be automatically managed by both the inhabitants and the remote users through a control center located on-site for access control to be implemented for authorized uses. The paper conceptualizes how the management system would integrate the subsystem in a smart district. The use of blockchain technology will store, send, and receive information through transactions in a peer-to-peer environment. The authors suggest that blockchain integration would enable application automation, and this technology would be a key element for increased cost competitiveness, leading to smart city deployment.

### 8.2. Blockchain-Enabled Federated Mechanisms

The authors in [58] propose a novel solution for distributed management of identity and authorization policies by leveraging blockchain technology to hold a global view of the security policies within the system and integrate it into the FIWARE platform. The authors aim to use the blockchain merely as a distributed data repository, leaving the distributed OAuth2-based implementation of the authentication and authorization logic external to the blockchain, as provided by FIWARE. It offers a rich set of open standard APIs to acquire data from the IoT of the smart city, process, store such data, and provide advanced user interaction. When such centralized management of policies is unsuitable due to the federated deployment applied to the system of interest or the multi-tenant model, more advanced solutions, such as a federation of databases, are needed. The performance assessment was achieved via blockchain using a federation of relational databases by employing a 3PC for guaranteeing consistency among multiple replicas. The mean latency of over 20 requests has been equal to about 390 ms with blockchain usage, while it was observed at 700 ms using a federated set of databases. The insert/update operations with the blockchain use were measured with 3160 ms and 2870 ms, respectively, and 50 ms and 30 ms using a federated database system. The results showed blockchain as a more beneficial technology when queried rather than implied for data management. The federated database system is faster as the distributed consensus is not needed.

### 8.3. Blockchain-Enabled IoT Embedded Assets

Recently a Blockchain-enabled solution has been proposed that utilizes Blockchain tokenization for asset identification by binding the tokens to the physical properties of the chip. The authors integrate Non-Fungible Tokens (NFTs) in [72] to represent assets by a unique identifier as a possession of an owner. The authors proposed a smart NFT that is physically bound to its IoT device. This mechanism also defines authentication mechanisms based on Physical Unclonable Functions (*PUFs*), which describe the physical properties of the devices and are used to identify and represent the devices using their private key and BCA address. They have a blockchain account (BCA) address to participate actively in blockchain transactions. These NFTs can establish secure communication channels with owners and users and operate dynamically with several modes associated with their token states. The authors demonstrated the proposal developed with ESP32-based IoT devices and presented the Ethereum blockchain, using the SRAM of the ESP32 microcontroller as the PUF.

### 8.4. Blockchain-Enabled E-Voting Mechanism in Smart City

Leveraging the Blockchain security mechanisms, the authors in [88] present the use case of the e-voting application of IoT, which is one of the prospective growth areas in technologies related to smart cities. The authors suggest blockchain-enabled solutions for the e-voting system to attain robust security and privacy and to discourage the problem of intruders performing rigging for the polls. The evaluation of the proposed mechanism was analyzed against several attack vectors in terms of message alteration, Denial-of-Service (DoS), Distributed Denial-of-Service (DDoS) attacks, and authentication delay.

### 8.5. Blockchain-Enabled Authentication Mechanisms

An Ethereum-based smart contract for edge computing has been proposed as SmartEdge in [89] for its low-cost, low-overhead tool for compute-resource management. The authors show the design breakdown of a smart contract into three key steps and describe them below in the context of their design of SmartEdge. Firstly, identify the parties involved in the smart contract, such as compute node (host the Ethereum emulator and the smart contract). Secondly, the data node will be responsible for sending/receiving data as defined in the smart contract, such as identifying key states in the lifetime of the smart contract. Thirdly, the five states are Unavailable, Available, Pending, Computing, and Completed, and identifying and defining the methods that trigger state transitions. The performance was evaluated in terms of low-overhead delay in executing a job and transaction cost in terms of costs that should not be significant relative to their value. Two factorization jobs were created to evaluate the overhead with input files consisting of 10,000 integers and 100,000 integers using the data node, compute node, and SmartEdge. This job roughly executes in 3 min on a Pi; however, when it was executed using SmartEdge, it only took 8.6 s. There is an overhead of 2 s compared to executing the job directly on the compute node. There was a noticeable 2-second overhead that included the time it takes to transfer the job to the compute node and the result back to the data node. The execution time of the larger input file was noted as 67 s on compute node, which shows increased latency in terms of larger input files which may affect the time for the Available and Completed states.

The authors in [90] proposed authentication and access control mechanisms based on a distributed architecture for lightweight IoT devices, which they claim, can imply many scenarios. The mechanism leverages the benefits of fog computing and public blockchain technologies, which provide a non-centralized medium since public blockchain is a non-centralized distributed ledger technology. The mechanism provides an initialization phase for registering a new IoT system and a device authentication phase for registering smart devices with blockchain fog nodes. The proposed mechanism provides a D2D communication phase for device communication within or for other systems and access control for IoT devices. The Elliptic Curve Digital Signature Algorithm (ECDSA) has been used for key generation, generating public and private keys for the devices and the fog nodes. The security requirements have been tested with the proposed mechanism: Confidentiality, Integrity, Identification, Non-Repudiation, Authentication, and Mutual Authentication. The evaluation was carried out in terms of execution time required by the IoT node for making the registration request (min: 1.06 ms and max: 1.25 ms) and the time needed by the node for sending a data message (min: 0.03 ms and max: 0.08 ms). Additionally, in terms of the CPU power consumed by the node for requesting registration (min: 7.24 mW and max: 10.32 mW) and power utilized by the node for sending a data message (min: 2.91 mW and max: 4.12 mW). A total of 100 experiments were carried out to evaluate the proposed mechanism, which shows promising results comparatively.

A proposed framework in [91] BCoT Sentry (Blockchain of Things Sentry) integrates blockchain with an IoT network. It enhances network security by analyzing network traffic flow patterns of the device obtained from data storage in the blockchain. The framework has been proposed to keep the lightweight feature of IoT devices which commonly fails to meet computationally intensive requirements for blockchain-based security models. (BCoT) Gateways are blockchain nodes where an IoT device security module is employed through a smart contract. These Gateways facilitate recording authentication transactions in a blockchain network; thus, the mechanism stores the device identity information in a distributed ledger. The authors present a novel approach to the feature selection method (similar feature selection method in machine learning utilizing the maximum information coefficient (MIC), used to measure the discrimination of IoT devices). It captures the IoT device traffic from the network layer and sends this traffic flow feature to the Smart Contract via blockchain transaction. The smart contract defines the device’s identity information and related operations and is triggered once the transactions in the blockchain are posted. The contract defines the access permissions policies that enforce the authorized access to modify or access the device identity information through a defined contract in the web3.py interface. The evaluation performance was measured in terms of device identification accuracy of detecting device identity fraud that exceeds 80%, and 21 of which exceed 90%. In terms of time complexity, 1000 calls were made to the functions *Register ()* and *Detective ()* on each BCoT Gateway and obtained the average response time. The identity authentication for the proposed IoT authentication model refers to Register and Fraud Detection. It has a time complexity of O (m × n) and O (m), considering the type of IoT device is ‘n.’ At the same time, when there are ‘m’ IoT devices.

A blockchain-based distributed authentication modeling scheme named BlockAuth has been proposed in [92]. The edge devices in the edge layer have been regarded as a node to form a blockchain network. The authentication scheme claims are suitable for password-based, certificate-based, biotechnology-based, and token-based authentication for high-level security requirement systems in Edge and IoT environments. A blockchain-based distributed authentication protocol has been developed using the blockchain’s consensus and smart contract capability. In contrast, a client-server-based approach has been adopted to deploy blockchain on the server machine, while the registration server and certificate issuing server have been deployed for user authentication and access control based on the certificate-based mechanism. BlockAuth Scheme was evaluated by the authentication time required to initiate the request to receive the result. The response time was tested for the centralized network and 4-peer, 6-peer, and 8-peer in the distributed network. The average response time of 4-peer, 6-peer, and 8-peer in two groups test for the passing scene was recorded as 2.24 s, 2.31 s, and 2.40 s, respectively, and for the failed scene was recorded as 2.22 s, 2.30 s, and 2.40 s, respectively. Comparatively, the average response time of the centralized authentication scheme is noted at 1.13 s, which has been significantly lower than the proposed scheme in terms of latency. It might be due to the network speed and consensus mechanism involved in the blockchain scenario. The authentication schemes have been deployed using the smart contracts, while claims for the biotechnology-based and token-based password authentication mechanisms have not been seen. PKI-based implementation in a client-server environment is prone to a single point of failure.

SSO (Single Sign-On) is a one-time password authentication scheme that requires a user to authenticate once, which helps avoid the fatigue of adding passwords again and again on the web. It includes a centralized approach with an authorized central body, such as a miner or server, which registers and issues a token for future access to various services and applications [93]. Alternate to SSO, the authors in [94] proposed a new Distributed Anonymous Multi-Factor Authentication (DAMFA) scheme that uses public blockchain (i.e., Bitcoin and Namecoin). The underlying consensus mechanism improves usability, which builds on a Threshold Oblivious Pseudorandom Function (TOPRF) for resistance to offline attacks. They claim to include a distributed transaction ledger technology such as blockchain to improve usability. It requires no interaction with the identity provider; hence, the user’s authentication no longer depends on a trusted third party. Namecoin blockchain is a public ledger blockchain that allows registering names and storing related values in the blockchain, a secure distributed shared database. The performance evaluation of the distributed anonymous authentication system has been carried out in two main steps: the registration and the authentication phases. The total time consumed in the registration phase for generating the credentials was noted at ≈703 ms, while the time consumed in the authentication phase for generating the credentials was noted at ≈640 ms. The results were achieved by running over 100 trials for the authentication and the registration phases.

A framework for the authentication mechanism based on blockchain has been proposed in [95] named BCTrust. It has been designed especially for devices with resource constraints such as computational, storage, and energy consumption constraints. Public blockchain Ethereum has been used together with C programming to deploy the mechanism to implement the framework. The robustness claimed by the authors is because of the underlying framework of the public blockchain, distributed ledger technology with no central authority for signing the contracts and principles known as smart contracts. These smart contracts provide access control over system (SID) authentication mechanisms and User or Device identification (UID). A practical implementation has been carried out on a network composed of two CPANs, while the performance evaluation of the proposed mechanism was measured in terms of execution time and power consumption of classical association and BCTrust association. The average time and power consumption of the BCTrust association were noted ≈ at 14,406 ms and ≈0.681 Joule, while that of a Classical association was noted ≈ at 34,450 ms and ≈2.755 Joule, respectively. It shows that BCTrust was comparatively more robust in terms of saving more than 75.28% of energy.

Blockchain-enabled fog nodes for user authentication schemes have been proposed in [96], which deploys smart contracts to authenticate users to access IoT devices. It is also used to maintain, register, and manage IoT devices, fog nodes, admins, and end-users. The fog nodes provide scalability to the system by relieving the IoT devices from carrying out heavy computation involving tasks related to authentication and communicating with the public blockchain. A distributed system based on the public blockchain design has been proposed with its implementation using Ethereum smart contracts for IoT device authentication at scale. The proposed Ethereum smart contract implements the authentication functionality for adding end-users and IoT assets with the help of an admin who takes care of the overall functionalities and operations of the authentication mechanism.

A proposal for IoT device authentication and identification utilizing Blockchain-based Internet of Things (IoT) Device to Device Authentication Protocol for Smart City Applications using 5G Technology (BIDAPSCA5G) has been presented in [97]. The authors deployed a private blockchain for the IoT device registration while the distributed ledger was utilized, storing device credentials to be accessed only by authenticated entities. The security analysis was performed against well-known attacks, showing promising results compared to the existing protocols. The authors in [98] proposed a Privacy-Preserving and Secure Framework (PPSF). It is a two-level privacy scheme consisting of an intrusion detection (ID) scheme based on a blockchain module and a Principal Component Analysis (PCA) technique. The first scheme is designed to securely transmit the IoT data while the PCA transforms raw IoT information into a new shape. Gradient Boosting Anomaly Detector (GBAD) was employed in the ID scheme for training and evaluation purposes. At the same time, a blockchain-InterPlanetary File System (IPFS) was integrated with Fog-Cloud architecture to deploy the proposed PPSF framework. Results were reported and compared with blockchain-based and non-blockchain-based solutions for comparison.

The authors in [99] propose an authorization system for IoT devices based on Blockchain. UDP (User Datagram Protocol) was chosen for the communication as it utilizes a simple communication model for nodes in the system. Encryption methods such as Vigenere cipher encryption was integrated to secure the communication, which are one-way hash functions for encrypting the data. The authors provide the mechanism to solve the security issues currently posed in centralized architectures.

A device management framework has been proposed in [100] to intercept the attacker’s intrusion through an unidentified device. The authors propose a Blockchain-based device management framework that progressively manages the known devices and provides resilience when the system is attacked in a smart city network. Smart contract manages the device management history that can be stored in Blockchain. The mechanism tracks the transmitting firmware between vendor and management node through a smart contract to attain robust security and resilience on the attack. The evaluation of the framework has been performed in terms of security services (i.e., confidentiality, availability, integrity, audit-ability, adaptability, and authentication).

The authors propose a lightweight data consensus algorithm based on blockchain technology in [101]. The authors focus on the Industrial Internet of Things (IIoT) to attain security in terms of data transmission in the IIoT for smart city applications. The algorithm utilizes edge gateways on a distributed ledger to achieve consistency in data transmission. The evaluation of the lightweight data block structure showed improvement over the traditional blockchain technology by reducing the average hop count of data transmission, thereby reducing the probability of data being stolen. The results were promising for achieving robust security with high data accuracy and reliability of the IIoT.

As mentioned previously, the blockchain-based authentication schemes review the distributed ledger technology (DLT) for IoT authentication in a distributed architecture; however, these schemes pose threats that the attack vector in cyberspace can exploit. Table 2 depicts a summary of distributed mechanisms with proposed authentication schemes, while the issues with those schemes have also been mentioned for future research challenges.

As stated in the previous sections, the blockchain-based authentication mechanisms depend on the copy of authentication requests distributed across all the nodes in a distributed architecture. This property makes it difficult for any possible breach; however, some of the authentication issues have been highlighted that need robust solutions and are discussed as under.

- The authentication and authorization solution have been proposed based on trusted third-party (TTP) distributed platforms such as FIWARE, which offers a rich set of open standard APIs to acquire data from the IoT of the smart city but not on the blockchain itself. In contrast, blockchain has been utilized merely as a distributed data repository.
  - The reliance on TTP distributed platform for authentication and authorization mechanism opens doors to adversaries on IoT-enabled smart devices.
  - The communication overheads (in terms of traffic, processing time, and energy consumption) are significantly higher than the base models concerning its security and privacy gains which would need to be considered in time-critical IoT applications.
- Different techniques can extract useful knowledge from big data by filtering, normalizing, and compressing IoT data. The IoT-enabled smart devices involve embedded devices, communication, and target services (blockchain, cloud); thus, savings in the amount of data that the IoT provides can benefit multiple layers.
  - A local storage device for backup data has been introduced in some of the proposed solutions whose security risks must be considered in authentication schemes open to attack vectors and may jeopardize the network security.
  - Smart contracts (SC) define applications that are distributed in nature and are special entities that provide real-world data in a trusted manner. The validation process of these smart contracts could be compromised since the IoT-enabled smart devices can be unbalanced.
  - SC in proposed solutions is not designed considering the heterogeneity and constraints present in the IoT-enabled smart devices in the smart city concept.
  - Functions and events in the SCs enable the actuation mechanisms to be employed directly on the IoT-enabled smart devices much faster.
  - Smart contract deployment with defined authentication functions may provide security, so authentication schemes with smart contacts/distributed apps (dApps) should be considered.
- The IoT-enabled smart devices have security issues from the manufacturer’s perspective as the asset’s firmware is not fully equipped with a security mechanism by default.
  - Especially authentication, access control schemes, and firmware updates are commonly found unattended, posing these assets’ exploitation.
  - Strong and lightweight encryption schemes such as one round cipher, etc., would help mitigate the authentication and access control issues based on communication and computational costs.
  - Running applications can be updated using partial upgrades, but the network stack must be updated by updating the firmware.
  - An effort has been made to update the firmware in run time, such as GITAR [103] and REMOWARE [104] architectures that support these assets in runtime for the network and firmware updates which is essential to ensure a secure integration of the IoT with blockchain over time.
- Heterogeneity among the assets is yet another issue at the network layer that poses a security threat. Many heterogeneous devices with weak or default security mechanisms operate, send, and receive data. At the same time, the adoption of BC for obvious reasons has proposed BC as a key technology to provide a much-needed security mechanism for IoT-enabled smart devices and the network.

As mentioned previously, two major sections presented a comparative review of authentication schemes based on centralized and distributed architectures, i.e., Section 7 and Section 8, respectively. Both of these architectures have been reviewed concerning the domains in the schemes employed, which converge the point of view to the future research challenges in the upcoming section.

## 9. Recent Advances and Future Research Challenges

This section presents the recent advances and the future challenges conceived from the review papers. In smart city infrastructure, the data is transmitted from multiple CPSs to the security operations center (SOC) over the internet, posing security threats in different communication architectures of the smart city. The security solutions need attention to build robust mechanisms that would eventually safeguard the IoT-enabled smart devices in a smart city concept. The below-mentioned recent advances with future research challenges in each section give an overview for future research in the fields of industry and academia.

### 9.1. Blockchain-as-a-Service (BaaS)

**Figure 5:**
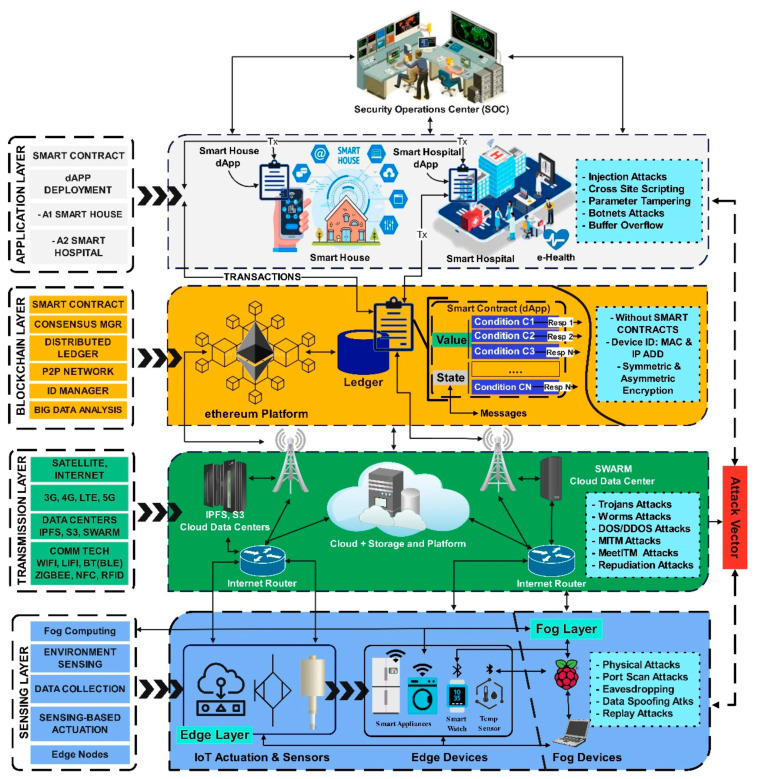
Blockchain-based Smart City Architecture.

As depicted in Figure 5 the blockchain-based layered architecture has been presented. It adds a BC layer to the generalized smart city layered architecture as presented in Figure 3 to integrate IoT-enabled smart devices in blockchain-enabled CPSs (such as smart homes, smart hospitals, etc.). The blockchain-enabled smart city architecture can be classified into four layers, while the inclusion of the blockchain layer supports robust security mechanisms. As stated in Section 2, the sensing layers deploy the edge and fog nodes (i.e., sensors, aggregators, and actuators) in the physical environment within cyberspace that supports actuation based on the data collection. Here, fog computing provides enough computational resources for data collection and processing for environmental sensing. The network provides connectivity using communication and transmission technologies at the transmission layer. In contrast, the command-and-control work on the application layer defines the applications for the asset’s behavior at the physical layer. As shown in Figure 5, the blockchain layer is of immense importance as it offers blockchain as a service (BaaS) in a smart city concept [105,106].

The underlying DLT and the consensus mechanisms provide robust security for communication that cannot be tempered. The posted data is shared among all the nodes in the BC network, making it distributed and in an immutable state.

This data cannot be altered unless and until the posted data is altered on all the distributed nodes, requiring a lot of processing and computational overhead. One main concern of the BC layer is to provide security services (Confidentiality, Integrity, Availability and Authentication, Authorization, and Audit) to the users and CPE (i.e., sensors and actuators) within CPSs in smart cities in a distributed manner. Apart from centralized architecture, distributed systems have also been in use traditionally. Still, the authentication mechanism for smart cities based on DLT is yet to be explored further for their use.

#### 9.1.1. Blockchain Tokenization

As shown in Figure 5, the BC layer opens many more opportunities to utilize BC-based services, such as blockchain-based tokenization schemes for asset identification and authentication schemes in smart city architecture. After a huge appreciation of Token creation in 2018, with over 1132 ICOs and STOs collecting nearly USD 20 billion [107], the concept of Token has gained wide attention. Tokenization in BC presents the concept of digital representation of an asset on the Blockchain or colloquially “programmable money”. There are different types of tokens presented by BC tokenization, tangible or intangible, such as security tokens, tokenized securities, utility tokens, and currency tokens (i.e., fungible or non-fungible) [108]. Tokens presented by BC tokenization are algorithms implemented as a Smart Contract on a Blockchain. CryptoKitties is one of the first-ever Ethereum-based collectibles game use cases that deployed tokens in a production environment, while other examples of collectibles are available for purchase on NFT marketplaces such as OpenSea [109], NBA Top Shot [110], etc. Since it maintains the data in a secure and immutable state, it attracted much attention, and a humongous amount of money has been. It is being invested in these virtual collectibles. Individual CryptoKitties are traded at over USD 100,000 [111]. One of the important aspects of the tokenization for stamps is determining the value by its rarity. That is how the SC algorithm guarantees uniqueness by mitigating the copies and limiting the maximal number of Tokens available. Ethereum platform has been used to generate Tokens through smart contracts. However, BC tokenization, such as non-fungible tokens (NFTs) leverages distributed networks through SC implementation, i.e., Ethereum implements the standard based on Ethereum request for comment (ERC-271 and ERC 1155) tokens specification.

#### 9.1.2. Non-Fungible Tokens (NFTs)

The ERC-721 standard defines guidelines for developing non-fungible tokens (NFTs) on the Ethereum blockchain utilizing smart contracts. Although the ERC-271 token has been defined under the category of currency tokens, these crypto tokens can be used apart for specified purposes. It can be used to identify and authenticate assets in a smart city infrastructure where a public key can identify users and devices can identify users and devices and transact uniquely by the identified tokens.

#### 9.1.3. Research Challenges in BaaS

The concept of Blockchain-as-a-Service (BaaS) has taken a huge appreciation as the use is not limited to cryptocurrency; rather, it has been expanded to multiple domains in the industry and academia. It increases the challenges for its deployment and integration in those domains. Mentioned below are the challenges that have been discussed from a future research challenges standpoint.

- Security Services: Weaknesses and Threats

Data integrity and availability are the issues with these assets that have to safeguard the huge amount of data that these assets generate. Data integrity and privacy are the key concerns that would help secure the data generated by the IoT-enabled smart devices; however lightweight cryptographic mechanisms are needed keeping in view the resourced-constraint nature of these assets. In case of compromised data integrity, if data uploads to the BC, it will stay corrupted as the data uploaded in BC remains immutable. It can identify its transformations, e.g., eavesdropping, denial-of-service or controlling the environment, participants, vandalism, the failure of the devices, etc.

- Anonymity and Data Privacy

Data anonymity is yet another challenge that can be achieved with data integrity and privacy by implementing distributed proxy re-encryption schemes. It would help the message be hidden until decoded by the recipient. Implementing distributed proxy re-encryption schemes together with BC would strengthen data anonymity. Trust is another key feature of the IoT where blockchain integration can play a role. Efficient and restricted access control for the IoT-enabled smart devices can be achieved by implementing data integrity techniques with an option to ensure data access simultaneously. It is preferable to avoid overloading the blockchain with the huge amount of data generated by the IoT.

- IoT-Enabled Assets Firmware Upgrade

Initiatives for firmware updates in run time would enable the network to have updated assets essential to ensure a secure integration of the IoT with blockchain over time.

- Storage Capacity and Scalability

Blockchain is not a medium for storing large amounts of data like those produced in IoT-enabled smart devices. Only useful data may be extracted from the humongous data generated by assets for extracting knowledge and making informed decisions, as in the case of actuation actions. Distributed storage platforms, such as an interplanetary file system (IPFS), Swarm, and S3, can be utilized. They can be integrated into the BC platform, as in the case of IPFS for Ethereum BC.

- Integration of IoT-Enabled Assets to Blockchain

As discussed in the review, the IoT integration in BC inherits the challenges as these IoT-enabled smart devices are resourced-constraints devices. At the same time, BC’s computational overhead for posting transactions causes integration issues. These devices also generate terabytes (TBs) of data in real-time, limiting their integration with blockchain.

- Smart Contracts

Overloading is an issue with the SC when accessing multiple data sources, the distributed nature of the SC would provide an edge; however, these SC can be expensive in terms of computation while processing huge computations. The process of filtering and group mechanisms may be incorporated into the SCs. It may enable applications to address the IoT-enabled smart devices depending on the context and requirements of the smart city concept. Interoperability among different cyber-physical systems in a smart city is another factor that needs SC deployments for overall assets and systems.

- Digital Representation of Assets

Another challenge is the device authentication and digital representation that has been achieved using traditional ways such as the device’s MAC or IP addresses. It exposes the devices with their embedded credentials in smart city networks from an adversarial point of view. However, blockchain tokenization can achieve it innovatively, especially with non-fungible tokens (NFTs). It can help mitigate device identification issues by representing and accessing the assets digitally with the help of smart contract functions and events.

### 9.2. Cryptosystems

**Figure 6:**
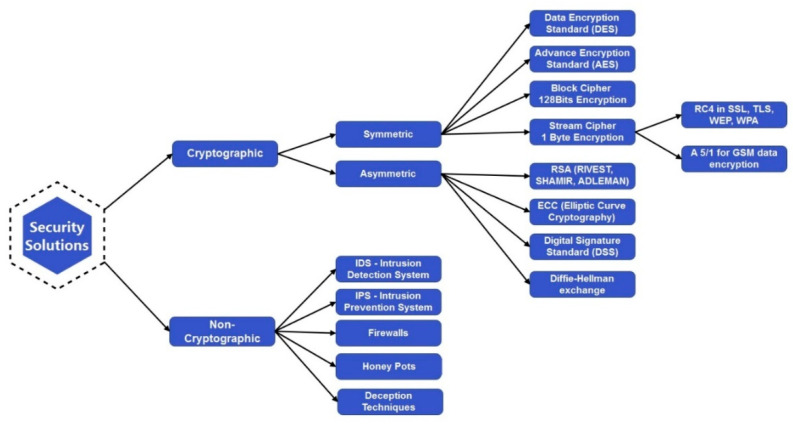
Security Solution based on Cryptosystems.

As shown in Figure 5, blockchain-based solutions have been proposed to provide security services (i.e., confidentiality, integrity, availability, and authentication schemes) for data utilizing cryptographic security schemes. It enables the system to attain robust security and privacy for connected parties and message exchanges. Blockchain-based solutions have opted for cryptographic schemes such as symmetric (such as DES, AES) and asymmetric (such as RSA, ECC, DSS, Diffie-Hellman exchange), which along with non-cryptographic solutions (such as IDS/IPS, Firewalls, and honeypots, etc.) as depicted in Figure 6. However, due to mathematical difficulty in solving the cryptographic hashes and the high communication payload, it has been a challenge to deploy these security schemes in CPSs for smart city infrastructure [81,89,96]. These schemes depend on the underlying PKI infrastructure of the blockchain or PKI-based implementation in a client-server environment or cloud for storing and managing assets. In contrast, the solutions based on centralized architectures widely employ cryptosystems to attain robust security and privacy, as discussed in Section 7.3.

#### 9.2.1. Research Challenges in Cryptosystems

The research to mitigate security challenges in smart cities mainly focused on authentication; however, in most existing authentication protocols, the trustworthiness for evaluating IoT-enabled smart devices in smart cities has been ignored. The authentication, authorization, and security services are of immense importance, which can be achieved by implying lightweight and robust cryptographic algorithms for securing communications.

- The new generation of cryptographic algorithms with low latency to generate the hashes has been introduced with one-round cipher algorithms. It utilizes the dynamic key approach. A dynamic key (that depends on a secret key and a nonce and generates different cipher text for the same plain text) is generated for each input, such as audio, image, or video. The proposed lightweight cipher algorithms are based on a dynamic structure with a single round of simple operations. They can help provide security for time-critical applications for resourced-constraints devices [112,113].

#### 9.2.2. Decentralized Key Management System

The new breed of cryptographic primitives needs to be explored based on distributed architectures such as decentralized key management systems (dKMS) that address the limitations of using consensus networks to store and manipulate private, encrypted data securely.

- Cryptosystems that are CCA (security against chosen-ciphertext attacks) secure, while notions of CPA-security (security against chosen-plaintext attacks) and CCA-security apply to proxy re-encryption.
- An example in this context is NuCypher, which enables sharing of sensitive data for distributed and centralized applications, providing security infrastructure for applications from healthcare to identity management to decentralized content marketplaces. It will be an essential part of distributed applications, just as SSL/TLS is essential for every secure web application; thus, security services based on distributed KMS need to be explored based on blockchain solutions [114].

## 10. Artificial Intelligence-Enabled Security Solutions

Artificial intelligence (AI) refers to the intelligence demonstrated by machines, with an idea perceived from the natural intelligence of humans and animals. It can be defined as any system that perceives its environment and takes actions to maximize its chance of achieving its goals [115]. Machine learning is a sub-class of AI that evolved in the first decades of the 21st century involving highly mathematical-statistical machine learning algorithms and has dominated the field. In machine learning, computer vision, speech recognition, speech processing, or optimization, AI makes it possible to gather the environment information through objects and provides informed decisions through processes in the physical world. Autonomous decisions can be made; thus, AI represents the brain of the digital world [111].

### 10.1. Artificial Intelligence-Enabled Blockchain-Based Security Solutions

AI-based mechanisms have been proven robust in mitigating authentication, access control, malware detection, and network security-related issues. However, a new breed of security mechanisms, i.e., distributed ledger technology, has been proposed to attain authentication robustness. Integration of blockchain technology to AI-based solutions may lead to mitigating security issues. For instance, a blockchain-enabled signature-based key management protocol in [116] has been proposed for Industrial Cyber-Physical Systems (ICPS). IoT smart devices can securely communicate with their respective gateway nodes (fog nodes). The key management system (KMS) is used as a protocol while blocks with secure data from smart devices by fog servers are presented to the cloud servers. These cloud servers then initiate the process of mining those blocks for verification and addition to the blockchain. The data in the mined BC in ICPS would be saved in a distributed manner, providing a more robust and protective system from a single point of failure, low latency, and cost-effective point of view. The AI-based machine learning techniques have been applied to process data on the blocks in blockchain for correct predictions that will be very useful for big data analytics.

The authors in [117] proposed a mechanism named Babelchain that provides a novel consensus protocol called Proof of Understanding (PoU). It aims to adapt Proof of Work (PoW) properties for IoT applications and suggests a system for integrating and designing a blockchain. It could learn as a training set for machine learning algorithms, more precisely logical ML algorithms, and supervised statistical models to suggest message formats. They discuss the implications of machine learning algorithms in a prediction model for M2M communication using the message formats by allowing the blockchain’s security aspect, i.e., Bitcoins Immutability Feature. A baseline mechanism for communication and security has been followed based on Bitcoin on top of the underlying mechanism. The intelligent machine learning algorithms have been applied to achieve the lightweight security named PoU (Proof of Understanding). The transaction logs posted to the blockchain contain all the *successful* or *unsuccessful* handshakes and a large list of relevant features. Processing transaction logs as training data sets to compare the tasks (i.e., applying supervised machine learning algorithm) by the Translator before the data is pushed to the blockchain that supports fulfilling the tasks. Tasks include creating a (statistical) model of machine message communication patterns, applying a predictive message format algorithm, and the impact of allowing to read which message formats can be useful for successfully closing handshakes.

The proposed authentication scheme in [118] utilizes unsupervised learning techniques to resolve nearby IoT-enabled smart device authentication issues. The authors practice a nonparametric Bayesian method, IGMM, to circumvent the “overfitting” problem. The model complexity has been adjusted to evaluate the RSSI and time of the packet arrival of the ambient radio signals to detect spoofers outside the proximity range. The performance evaluation shows promising results as the proposed authentication scheme has been noted with decreased the detection error rate by 20% to 5%, with those implying the Euclidean distance-based authentication in the spoofing detection experiments for indoor setups.

#### 10.1.1. Machine Learning

Machine learning is the branch of artificial intelligence that helps the machine learn from the data with the help of the algorithms that process and produce the knowledge from the data. Machine learning can be classified into supervised, unsupervised (ensemble learning), semi-supervised, reinforcement, and deep learning that covers a broader range of techniques such as clustering, classification, prediction, estimation, etc., depending on the nature of data [119]. It can help accomplish the most common tasks such as prediction (regression) and classification [3]. Much research has been carried out in the network security domain where the hosts are fed with malicious codes in the form of malware to detect distributed denial-of-service (DDoS) to chock the system, for face and character recognition, etc. [3]. The security of data from the cyberattacks such as distributed DoS (DDoS) attacks, network intrusions, DoS attacks, spoofing attacks, jamming, malware, man-in-the-middle (MITM), eavesdropping, etc., has become inevitable [2,27,56]. While this class of AI has proved highly successful and robust in solving many challenging problems, it comes with a problem of requiring huge data sets for analysis, computational overhead, and time for analyzing the patterns within the data. However, it is widely accepted throughout the industry and academia [115].

On the other hand, authentication schemes based on ML techniques have also been proposed, focusing on the physical layer features. The media access control (MAC) address, received signal strength indication (RSSI), and time interval (∆T) of packets received at a specific time duration are discussed in the next section. Thus, the researchers focus on ML techniques for AI-based solutions.

- Machine Learning for Authentication of IoT-Enabled Smart Devices

Machine learning for IoT-enabled smart devices has been an important tool for authentication issues and to protect against cyberattacks. These smart devices depend on technologies such as Radio-Frequency Identifications (RFIDs), Bluetooth (BT), Wireless Fidelity (WiFi), Wireless Sensor Networks (WSNs), Cloud Computing (CC), Fog Computing (FC), etc. Hence, researchers have utilized the physical features from network layers, such as RSSI of WiFi signals, radio signals, etc., to fulfill the need for the security and privacy of the devices.

The authors in [120] proposed a lightweight, intelligent authentication approach. It utilizes the ML technique, i.e., support vector machines, at the gateway to identify IoT-enabled smart devices’ access time slots or frequencies. The scheme has devised a mechanism to analyze the complex dynamic environment and achieve adaptive access control. This ML-based AI architecture supports the link between transceivers quickly. It enhances security instantly and helps mitigate the communication latency and security risks that are well-controlled in large-scale IoT infrastructures such as smart cities.

The authors in [121] utilize the physical layer attributes for authentication schemes and evaluate the security performance of key-less authentication schemes. Machine learning techniques have been utilized in the same scenario exploiting different one-class nearest neighbor (OCNN) classification algorithms. The evaluation of the authentication scheme exhibits a low probability of missed detection under the same probability of false alarm by deploying one-class classification (OCC) algorithms when a low spatial correlation exists between the main channel and the adversary.

Utilizing the physical layer attributes in [122], the authors proposed the authentication scheme that exploits the RSSI when received by multiple landmarks. Logistic regression was applied to avoid being restricted to a known radio channel model. dFW and IAG algorithms were exploited to estimate the parameters of the logistic regression model that have been proven inexpensive regarding the communication overhead. Additionally, progression has been observed for spoofing detection accuracy. The evaluation of the scheme showed that the average error rates of the dFW-based authentication and the IAG-based scheme are 6% and less than 10.4% espectively. IAG presented much better results in terms of communication overhead as it reduced the overhead by 73%, while dFW reduced it by 37.4%, compared to the Frank-Wolfe-based scheme.

- Machine Learning and Deep Learning-Based Solutions

The implication of existing ML and DL solutions for addressing different security problems in IoT-enabled smart devices and networks as far as network intrusion detection (NIDs) are concerned been discussed in detail in [2]. In contrast, the authors reviewed the authentication models and attacked vectors in [28] using machine-learning techniques for IoT security solutions based on supervised, unsupervised, and reinforcement learning (RL). ML-based IoT-enabled smart device authentication, access control, and malware detection schemes to protect data privacy provide insight into useful and robust security measures to improve network security. The techniques such as support vector machines (SVMs), naive Bayes, K-nearest neighbor (K-NN), neural networks (NNs), deep NNs (DNNs), and random forest have proved to be beneficial in the authentication process and restricting the attack vector based on the classification or regression model. Security mechanisms based on artificial intelligence (AI) also enable security provisioning approaches to achieve fast authentication and progressive authorization.

IoT-enabled smart devices have been tested with deep-learning techniques such as DNNs to refine the authentication accuracy. The DNN-based user authentication has been presented in [123] that focuses on exploiting the Wi-Fi signals’ channel state information (CSI) features, excluding the authentication dependency on assets. The proposed mechanism utilizes human behavioral and physiological characteristics extracted from their daily activities, such as stationery and walking patterns, to develop the DNNs model. The model effectively implements an authentication scheme for authenticating the user and identifying them as legitimate to mitigate the attack vector such as spoofing attackers. The scheme was noted with a user identification accuracy of about 94% for walking and 91% for stationary users.

On the other hand, ML and DL have been extensively utilized in intrusion detection and prevention systems (ID/PS). The authors in [124] discuss detecting intrusions, e.g., IDs, man-in-the-middle (MITM) attacks, etc., to possibly predict the invalid attributes which have been proved useful. The data was collected in terms of Device ID, Sensor Value, and Delay Time. The change in data generation patterns made the Artificial Neural Networks (ANNs) algorithm ring the alarm for false attributes generated by interfering IoT devices. The samples were compared for decision (True positives) making purposes so that the ANN model accurately predicted with 99% accuracy.

#### 10.1.2. Tiny Machine Learning and Deep Learning

As mentioned in previous sections, machine learning (ML) and deep learning (DL) are considered expensive in terms of latency and processing performance and need high-end hardware, with training and inference at the edge executed by gateways, edge servers, or data centers. However, due to industry and academia’s intensive research and development, distributing computational resources between the cloud and the edge has moved to the evolution of artificial intelligence (AI) in embedded assets. The assets with the latest microcontrollers with embedded ML accelerators have been made capable of delivering many Trillions of Operations per Second (TOPS) at the edge of the IoT infrastructures obsoleting high-performance processors to perform ML. A model optimization toolkit named TensorFlow is used for deploying, executing, and optimizing ML models. It supports latency reduction and model implementation to IoT-enabled smart devices with resource constraints in processing, memory, power consumption, network usage, model storage space, and optimizing existing hardware or new special-purpose accelerators. The tiny machine learning and deep learning models have extensively been used in IoT-enabled smart assets for user authentication schemes, especially in automotive and mobile devices such as biometric authentication and voice recognition for user authentication.

Similarly, the authors proposed a user authentication scheme in [125] that implements touch dynamics. The scheme utilizes a set of behavioral features to identify accurate user authentication. The performance evaluation was carried out on collected touch gesture data of 20 Android phone users. It was compared with several known machine learning classifiers—the average error rate of about 7.8% for selected features was observed, which shows that a neural network classifier is well-suited to authenticate different users.

On the other hand, tiny ML/DL has opened doors to be exploited in a different context, i.e., model deployment to IoT-enabled smart devices for user authentication using computer vision. For instance, the authors in [126] have proposed full development for a face recognition model supported by a live QVGA camera on RISC-V MCU with 512 kilobytes of internal RAM. The main application architecture consists of four stages, i.e., frame capture, face detection, face recognition, and user interaction (e.g., displaying message) in terms of the actuation process. It enables the IoT-enabled smart devices to deploy the DNN models and interpretation in extreme environments and the display preview on a battery-powered board for user authentication.

#### 10.1.3. Research Challenges in Artificial Intelligence

There have been many advances and deployments in network security through ML-based solutions such as IDS/IPS systems for intrusion detection. These solutions provided unmatched security but at the cost of high computational cost, processing overhead, and high latency. However, the algorithms have evolved into highly mathematical-statistical machine learning algorithms yet have dominated the industry. The AI-enabled BC-based solutions have also been proposed, which combine unmatched and robust security features utilizing fog computing. This space is growing quickly and will become a new and important application of artificial intelligence in the industry within the coming years. Yet, the challenges to be explored are high computational cost, processing overhead, and high latency.

- Tiny Machine Learning for IoT-Enabled Smart Devices

Advancements in tiny machine learning have led researchers to develop ML-based models that are more energy-efficient and new intelligence based on data-driven algorithms. They can be deployed on microcontrollers to operate sensors and actuators for preventative maintenance of surveillance cameras in smart city architecture. The issues relating to latency, power consumption, and data transfer (as and when required) have given the Tiny Learning Techniques (TLT) a new direction to perform these functions at low cost and with very low power consumption. However, mentioned below are the issues that pose challenges in this arena.

- Efficient Resource Allocation for IoT-Enabled Assets

In a nutshell, IoT-enabled assets with the latest microcontrollers embedded with integrated ML accelerators would strengthen the representation of bringing computing to sensors such as microphones, cameras, and those monitoring environmental conditions that process the data for actuation in smart city concepts. The current ML/DL-based mechanisms for IoT-enabled smart device authentication and access control have been explored in profiling assets in terms of their hardware imperfections. Thus, a need to explore additional information for improved device profiling must be considered for IoT mechanisms’ robust and high-end performance. However, challenges such as user authentication based on additional device information, more memory, low computational costs, low latency, and energy-efficient machine learning algorithms are yet to be explored.

- Digital Keywords Improvement

Other challenges need improvements in “keyword spotting,” such as Apple’s Siri and Google’s Assistant, and “visual wake words,” such as binary classification of an image that would mark it as a present or not present.

- Data Pruning for Tiny ML-Based Solutions

Data collection and processing are crucial to test and train the ML-based solution, as are the “trim insignificant weights” techniques for tiny ML-based solutions. Though 6× improvements in model compression with minimal loss of accuracy have already been achieved yet, improvements in latency are a challenge that the framework support would provide.

## 11. Conclusions

This paper provides an updated literature review of proposed authentication schemes in the IoT context for smart cities. The review poses a large spectrum of authentication schemes that identified many requirements and open issues to be considered by the researchers to develop robust, lightweight schemes. A categorical approach presents the centralized and distributed architectures for IoT-enabled smart assets that pose threats and need consideration as far as a security standpoint in smart cities is concerned. Considering the resourced-constraint nature of the low-powered IoT-enabled smart assets for the smart city infrastructure, blockchain (BC)-based solutions and distributed algorithms must be explored as most smart city deployments are centralized. It poses threats from a single point of failure and a single point of contact from a device authentication perspective. The BC-based solution has issues storing data generated by the assets for which the distributed storage platforms, such as an interplanetary file system (IPFS), Swarm, S3, etc., may be explored. This integration may support storing data hashes to avoid storage exhaustion issues.

A new generation of cryptographic algorithms needs to be developed and deployed to attain robust security services such as data and device anonymity and integrity. The performance evaluation of the new generation of cryptographic algorithms with low latency to generate the hashes should be explored. It will help provide security for time-critical applications keeping in view the resourced-constraints nature of IoT-enabled smart devices. Decentralized key management systems (dKMS) have to be explored in this context to address the limitations of using consensus networks for securely storing and manipulating private, encrypted data can be considered.

Machine learning approaches for IoT-enabled smart devices such as deep learning and reinforcement learning can be explored for smart city-state estimation in a data-driven fashion. Tiny ML/DL models must be developed to support authentication schemes for IoT-enabled smart devices. Data storage issues can be handled within real-time data collection, analysis, and decisiFons. It may help in continuous system monitoring to avoid unnecessary information for storage. The identified security issues have been categorized based on authentication architecture and discussed, providing future research challenges accordingly.

## Figures and Tables

**Table 1:** IoT-Enabled Smart Device Authentication Schemes based on Centralized Architecture for Smart City.

RP	Solution	Scheme	Issues
[57]	Smart Home-based IoT assets evaluation	- Systematized the existing literature for home-based IoT devices through an abstract model to derive insights. - Evaluation of IoT-enabled smart devices has been carried out and found issues related to IoT assets misconfiguration, weak authentication, vendors patch through device updates, etc.	- The methodology poses a centralized architecture based on cloud computing utilizing cloud endpoints for IoT assets communication, storage, and security issues. - Authentication, authorization, and security services such as confidentiality, integrity, and availability have not been discussed.
[42]	Hardware-Assisted PKI infrastructure	- The proposed hardware-assisted (TPM 2.0) framework provides the security services such as the confidentiality and integrity of data between each node and provides authentication for the communicating parties. - Furthermore, the presented framework is resistant to software-based remote hijacking as well as secret key extraction.	- The framework is hardware-assisted; hence, an upgrade is required, which needs the manufacturer’s intervention and can be costly to implement. - The framework implements Certification Authority (CA)-based PKI infrastructure, which poses security threats based on centralized architecture for communication between the user and the power grid network.
[43]	PUF-based authentication mechanism	- Two-factor authentication schemes for edge nodes using hardware upgrade. - The SRAM-based physically unclonable functions have been used to generate unique digital fingerprints of the device, which is used as the device Physically Unclonable Functions (PUFs) - Physical (attacks) damage control can be achieved for IoT devices vulnerable to internal and external attack vectors.	- Hardware upgrade is required, which needs the manufacturer’s intervention and can be costly to implement. - PUFs result from the manufacturing process of Integrated Circuits (ICs), which introduces random physical variations into the microstructure of an IC, making it unique. - PUFs utilize the SRAM of the edge node, which increases the operational and processing overhead resulting in delayed operations. - Single point of failure and contact (Attack Vector).
[68]	TBLUA	- Lightweight token-based authentication mechanism for Smart Hotel or Smart Office. - Central Reservation Server (RS) is responsible for generating tokens for users and registration authority (RA) which further manages to register GW and devices.	- Single point of failure and contact (Attack Vector). - Reg. server (RS) is responsible for generating tokens on the internet in a centralized architecture which causes security and data privacy issues. The client-Server environment is prone to spoofing attacks with the exposed share session key
[71]	RSA Authentication for Smart IoT	- RSA-based authentication system for Smart IoT assets has been designed. - The scheme provides security services, including X.509 certificate, RSA-based Public Key Infrastructure (PKI), and challenge/response protocols with the help of proxy-induced security service providers. - Applications for other multiple sensitive apps such as smart city apps, cyber-physical systems, etc. have been implemented, which needs the support of X.509 certificate based on hard tokens to populate other security services, including CIA	- Scheme is dependent on traditional PKI infrastructure, which is dependent on the centrally controlled architecture of CA authority. - RSA cryptosystem for authentication requires processing overhead, which is unsuitable for resourced-constraints IoT assets with the number of authentication requests. - IoT smart devices are very limited; hence, RSA authentication will be difficult even with a few authentication requests.
[75]	Smart Offices ML-based Authentication Schemes	- Machine Learning techniques have been utilized to classify the users according to their behaviors and authenticate users. - Cloud computing paradigm has been utilized to develop the architecture that implements the ML techniques. - The scope is limited to only Machine Learning (ML) algorithms such as Random Forest, which has been used to generate the models for classifying users.	- The validation of a higher number of users has been ignored, which may provide insights into detection issues to achieve robust solution goals. - Other ML algorithms, such as anomaly detection systems, must have been tested for comparison. - Latency and processing issues have not been taken care of. - Authentication and security services have been ignored.
[76]	Smart Home-based Device Identification Schemes	- iVision system has been designed and implemented on SoC (System on Chip) to achieve higher performance and less latency. - One of the main application usages of the iVision hardware solution is for hand tracking with the help of a depth map generated by the stereoscopic camera. - Smart home appliances are implemented based on DPWS (Device Profile for Web Services). - Additionally, context-awareness-driven architecture was suggested to improve the quality of the service in a Smart Home system.	- The analysis shows performance and increased latency issues in adding and probing more devices. - If two devices are registered in-network with the same web service, the DPWS requires a longer time or even fails to detect any devices in the network. - Lots of probing messages for device identification made the network behave peculiar. - Authentication, authorization, and security services such as confidentiality, integrity, and availability have not been discussed.
[77]	PUF-based authentication mechanism	- Mutual authentication schemes for edge nodes using hardware upgrade. - Physical damage control can be achieved for IoT devices vulnerable to internal and external attack vectors. - PUFs result from the manufacturing process of Integrated Circuits (ICs), which introduces random physical variations into the microstructure of an IC, making it unique.	- Hardware upgrade is required, which needs the manufacturer’s intervention and can be costly to implement. - PUFs utilize the SRAM of the edge node, which increases the operational and processing overhead resulting in delayed operations. - Single point of failure and contact (Attack Vector).
[78]	RSA Authentication for IoT	- An evaluation has been performed to deploy RSA authentication by limiting CPU cycles to simulate a low-performance device. - Evaluation involved a series of tests on this device running on low CPU cycles shows that it is impossible to run RSA authentication in the provided scenario.	- RSA cryptosystem for authentication requires processing overhead, which is unsuitable for resourced-constraints IoT assets with the number of authentication requests. - IoT smart devices are very limited; hence, RSA authentication will be difficult even with a few authentication requests. - Additional security requirements such as bad password handling by users and comparison with another cryptosystem, i.e., ECC, are missing.
[79]	E-Governance XOR and hash-based operations	- A central server-based XOR and hash operations for the password, smart card, User anonymity, Mutual authentication, shared session key, and Key freshness have been used. - The mechanisms are Smart Card (SC) dependent, as the public, private, and session keys are stored for registration, login, and authentication. - Sharing session keys using public and private keys infrastructure.	- The architecture is based on a centralized architecture that depends on the central machine for storing and creating passwords and sharing the session keys for mutual authentication and other security operations. - It is a single point of failure and contact from the attack vector perspective, as the scheme depends on a centralized server to generate the public, private, and session keys.
[80]	E-Governance	- The communication between the applications and the smart city is carried out using an advanced multi-factor user authentication scheme which can be utilized for the smart e-governance applications in smart cities. - The registered users receive a valid smart card accumulated with some parameters as confidential values needed to login into the system and contact the registered cloud server to avail themselves of the services.	- The mechanism is centralized and mainly depends on the central registration center (RC) that provides the parameters to the participants during the registration phase. - In case of compromised session keys at RC, the whole system would be at risk of being attacked
[81]	Data-Centric Edge Computing	- A data-centric edge-computing infrastructure defines the defense mechanisms to be hosted in IoT clouds by integrating physical states in decentralized power-grid regions and not power grid IT networks.	- The risk of compromised communication between the corporate network of the power grid and the edge server deployed at the cloud via the internet. - Identification and authentication of devices and the system have not been taken care of, as in case of an adversary, the power grid system behind the corporate network would be at risk. - It would act as a single point of contact for the attack vector while security policies at the edge nodes to command permissions control only (i.e., “read” and “execute” permissions) poses a strong threat.

**Table 2:** IoT-Enabled Smart Device Authentication Schemes based on Distributed Architecture for Smart City.

RP	Solution	Scheme	Issues
[51]	The Case Study of a Smart Home.	- Fundamental security traits, i.e., Confidentiality, Integrity, and Availability (CIA), has been achieved in a Smart home model by integrating the blockchain-based solution.	- The communication overheads (in terms of traffic, processing time, and energy consumption) are significantly higher than the base models concerning security and privacy gains. - Local storage device for backup data has been introduced, which is open to attack vectors and may jeopardize the network security.
[58]	Federated BC-based Solution Hyperledger Fabric 1.4	- A solution for distributed management of identity and authorization policies based on a decentralized OAuth2-based authentication and authorization solution utilizing the FIWARE platform has been adopted for realizing a smart city. - Hyperledger fabric 1.4 blockchain has been utilized to secure data in a distributed manner using distributed ledger technology.	- The authentication and authorization solution have been proposed based on a distributed FIWARE platform and not on the blockchain itself. - Hyper fabric blockchain has been used merely as a distributed data repository. - No smart contacts/distributed apps (dApps) deployment.
[72]	NFT-based authentication mechanism utilizing PUF	- Proposal of Non-Fungible Tokens (NFTs) has been utilized to bound the IoT assets physically employing PUFs. - Physically Unclonable Functions (*PUFs*), have been utilized as a low-cost solution to identify devices for solutions implemented on the blockchain. - NFTs represent assets by a unique identifier as a possession of an owner. - NFTs were bound to the IoT asset’s physical properties utilizing added PUF hardware.	- The proposal is hardware-dependent, requiring a hardware upgrade from the manufacturer, which may incur manufacturing costs. - With hardware upgrades, the IoT assets have been noticed to have increased initialization time, which incurs latency issues such as initializing Bootloader, located in the main SoC’s internal OTP memory. - The coding of the Bootloader cannot be modified since it is the device’s Root of Trust (RoT).
[88]	E-Voting in Smart Cities	- Proposal of Blockchain-based E-Voting mechanism for Smart Cities. - To attain high-end security and privacy to discourage rigging in polls. - The mechanism has been presented with security evaluation in terms of message alteration, Denial-of-Service (DoS), Distributed Denial-of-Service (DDoS) attacks, and authentication delay.	- Security data services such as confidentiality and availability concerns have not been taken care of. - Latency in terms of time complexity is missing as it is crucial for time-critical solutions. - Depended on Blockchain PKI infrastructure-based implementation.
[89]	SmartEdge-Ethereum	- SmartEdge, an Ethereum-based smart contract for edge computing, has been utilized, showing that the solution is a low-cost, low-overhead tool for compute-resource management. - The smart contract has been used to define and deploy the design in three key steps such as, - Identifying the nodes, - Identifying the functions for key states of the nodes, and - Defining the methods that trigger state transitions.	- Identification and authentication of devices have not been taken care of as in the malicious node, the proposed system would be at risk. - No results verification has been involved in verifying that a job was properly performed through the smart contract or not, and - Auctioning contracts such as automatically matching data nodes with the most appropriate compute nodes have not been taken care of. - The communication between the edge node and the distributed platform using the internet poses a threat to the whole system.
[90]	BC-based authentication mechanism Ethereum	- Authentication and access control mechanisms for edge devices in an IoT system. - Interoperability among the fog nodes of different IoT systems to operate in smart city infrastructure.	- Security and privacy issues are depended on Blockchain PKI-based implementation. - Security data services such as confidentiality and availability concerns have not been taken care of.
[91]	BCoT Sentry-Ethereum	- A network security module has been deployed, which analyzes the traffic flow of IoT devices and sends it to Smart Contract. - The maximal Information Coefficient (MIC) method has been used for feature extraction from the device traffic flows, which is used for device identification.	- Security and privacy issues are depended on Blockchain PKI-based implementation.
[92]	BlockAuth	- The Client-Server approach has been adopted to deploy blockchain on the server machine. - The registration and certificate issuing servers have been deployed for user authentication and access control based on a certificate-based security mechanism. - Edge devices build blockchain nodes and provide a distributed, safe, reliable solution.	- Client-Server-based approach has opted for certificate issuance, while communication between RS and edge nodes has not been taken care of. - Claims for the biotechnology-based and token-based password authentication mechanisms have not been seen. - PKI-based implementation in a client-server environment is prone to a single point of failure.
[94]	DAMFA-Bitcoin and Namecoin	- Blockchain technology to improve usability builds on a Threshold Oblivious Pseudorandom Function (TOPRF) to improve resistance to offline attacks. - Claim to improve usability without any interaction with the identity provider. - The trusted third party is no longer needed for user’s authentication.	- Depended on Blockchain PKI infrastructure-based implementation.
[95]	BCTrust-Ethereum	- Interoperability of different node registration in different CPANs has been realized by integrating blockchain (Ethereum), and an evaluation of the proposed mechanism has been carried out.	- Access control and Mutual authentication of sensor nodes are missing. - Depended on Blockchain PKI infrastructure-based implementation.
[96]	User Authentication using Fog Nodes	- User authentication mechanism for accessing IoT devices using fog nodes. - Fog nodes have been used as Blockchain nodes. - Blockchain-based authentication system.	- Interoperability among the fog nodes in an IoT System is missing. - Data integrity and repudiation security issues have not been effectively handled.
[102]	Smart District Model	- The design of a smart district model has been proposed to build a smart city using blockchain and smart contracts to achieve an efficient energy management system, including energy, security, safety, environmental management, communication, information, etc.	- The mechanism discusses the basic properties of blockchain technology using smart contracts but does not consider confidentiality, integrity, privacy, and availability. - Challenges about cyber-physical systems in a smart city concept.
[97]	BIDAPSCA5G for Smart Cities	- A proposal for IoT device authentication and identification. - Blockchain-based Internet of Things (IoT) Device to Device Authentication Protocol for Smart City Applications using 5G Technology	- Smart contracts integration can improve the security services by limiting the IoT device availability to unauthorized users, thus increasing confidentiality, which has been missing in the proposal. - Depended on Blockchain PKI infrastructure-based implementation.
[98]	PPSF for Smart Cities	- Privacy-Preserving and Secure Framework for intrusion detection utilizing Blockchain module and Machine Learning techniques. - A two-level privacy scheme consists of an intrusion detection (ID) scheme based on a blockchain module and a Principal Component Analysis (PCA) technique.	- Ethereum platform has been utilized with the traditional Proof of Work (PoW) consensus mechanism, which poses performance issues of fault tolerance, decentralization, stability, and high-level security. - PoW also poses an energy consumption problem, which is unsuitable for smart city solutions. - Latency in terms of time complexity is missing as it is crucial for time-critical solutions.
[99]	Authentication System for IoT Devices	- A Proposal for an authorization system for IoT devices based on Blockchain. - Lightweight communication protocol UDP has been integrated to deploy a simple communication model for lightweight IoT devices. - Integration of the Vigenere cipher encryption method makes the scheme robust in providing data integrity over the blockchain network.	- Smart contracts integration can improve the security services by limiting the IoT device availability to unauthorized users, thus increasing confidentiality, which has been missing in the proposal. - Personal BC based on Python was developed, lacking many key security features; hence the proposed solution lacks a public or private BC network deployment and evaluation. - UDP has been employed to achieve the lightweight communication mechanism for IoT devices which may result in data packet loss with no resilience property defined.
[100]	Device Mgmt Framework	- A Proposal of a Blockchain-based device management framework for device identification. - Smart Contract has been utilized to manage the device history in Blockchain for known devices. - The proof of stake (PoS) consensus mechanism has been utilized, which is efficient in energy consumption. - Framework ensures Security services such as confidentiality, availability, integrity, audit-ability, adaptability, and authentication.	- The proposed framework uses a private blockchain, and latency has been judged to have higher transaction bandwidth in transaction processing but, - Latency in terms of time complexity is missing as it is crucial for time-critical solutions. - Depended on Blockchain PKI infrastructure-based implementation.
[101]	Security Schemes for IIoT	- A Proposal of a lightweight data consensus algorithm based on blockchain technology for IIoT. - Edge layer has been utilized to achieve consistency in data transmission over a distributed ledger. - The BC technology benefits the scheme by reducing the average hop count of data transmission resulting in no data spoofing.	- Latency in terms of time complexity is missing as it is crucial for time-critical solutions. - Depended on Blockchain PKI infrastructure-based implementation.

## Data Availability

The data used to support the findings of this study are available from the corresponding author upon request.
